# Effectiveness of public health measures and strategies to reduce risk of spread of respiratory pathogens at sporting mass gatherings: systematic literature review

**DOI:** 10.3389/fpubh.2026.1789413

**Published:** 2026-04-08

**Authors:** Lucia Mullen, Amanda Kobokovich Mui, Crystal Watson, David Heymann, Brian McCloskey, Gwenda Hughes, Chris Bonell

**Affiliations:** 1 Public Health, Environments and Society, London School of Hygiene & Tropical Medicine, London, United Kingdom; 2 Johns Hopkins University Center for Health Security, Baltimore, MD, United States; 3 Department of Environmental Health and Engineering, Johns Hopkins Bloomberg School of Public Health, Baltimore, MD, United States; 4 Epidemiology of Infectious Diseases, London School of Hygiene & Tropical Medicine, London, United Kingdom; 5 Global Health Programme, Chatham House, London, United Kingdom; 6 Department of Infectious Disease Epidemiology, London School of Hygiene & Tropical Medicine, London, United Kingdom

**Keywords:** COVID–OVI mass gathering, olympics, paralympics, respiratory illness, sports, systematic literature review (SLR)

## Abstract

**Objectives:**

To determine the type and effectiveness of public health interventions implemented at sporting mass gatherings to mitigate respiratory infectious disease spread and understand how feasible and acceptable the interventions were to implement.

**Design:**

Systematic review.

**Data sources:**

Medline, EMBASE, Cochrane Library, Scopus, Web of Science, Global Health, Epistemonikos, Global Index Medicus, WHO Library, WHO IRIS, IOC and FIFA were search in June 2023 and July 2025.

**Eligibility criteria for selecting studies:**

Studies that assessed public health strategies for sporting mass gatherings aiming to reduced respiratory infections were included. Publications prior to 2000, predictive modeling studies, commentaries, editorials, literature reviews, pre-prints and studies that did not retrospectively discuss official sporting events were excluded.

**Results:**

Thirty-four articles assessing 37 sporting MGs were included. The most common MGs assessed were the Olympic Games (*n* = 10). Almost all articles described multi-layered intervention packages including bubble approaches, routine testing, country entry screening, masking, physical distancing and/or isolation and quarantine. Based on an effectiveness framework developed for this study, 23 articles described effective intervention packages, three described non-effective packages and six were indeterminate. Feasibility concerns appeared a challenge for MGs with many spectators and linked to scalability issues. Acceptability factors were likely influenced by perceptions of increased work burden, compliance levels and stakeholder engagement.

**Conclusion:**

This systematic review provides the first opportunity to comprehensively map pre-pandemic and pandemic-era planning for sporting MGs and underscores the importance of multilayered, context-specific intervention packages which may meaningfully reduce the risk of respiratory disease spread.

**Systematic Review Registration:**

CRD42023433619.

## Introduction

Mass gatherings (MGs) are defined as events bringing together many people with the potential to strain local health-system resources. Importantly, WHO does not provide a size limit to define a MG as this number can vary depending on the existing health system and available resources in the host country (1). Due to the nature of MGs, specifically the large concentration of people in close interaction, the risk of respiratory disease spread can be high (2–4). However, MGs can provide positive economic impact felt across communities (5, 6). Participating in sporting events also provides social wellbeing benefits and opportunities for social support. The benefits of sporting MGs underline their importance and necessitate the need to develop comprehensive safety protocols that focus on reducing the risk of infectious disease spread while allowing events to occur with minimal interruptions (7). Understanding the public health mitigations available is vital to improve overarching MG preparedness and reduce the potential health burden on the host country and spread to other countries (1, 8, 9).

Various public health interventions have been used during sporting MGs both prior to and during the COVID-19 pandemic to minimize respiratory infections (1, 2, 8–10). However, there is limited attention paid to this field. The majority of available systematic and non-systematic reviews are focused on religious pilgrimages (11–15). Others primarily examine types of respiratory diseases or threats reported and cover MGs broadly (16–19). Of the existing sporting-specific reviews, three are scoping reviews on risk factors for infectious disease generally and include some focus on prevention measures or recommendations for pre-, during, and post-event implementation to mitigate disease spread (20–22). Another systematic review considers the effectiveness of one intervention, digital surveillance systems, at sporting MGs (23). Only two reviews are available that examines a range of infection prevention and control strategies but look at MGs broadly rather than solely sporting events. One is a systematic review that examines respiratory pathogens with pandemic potential at MGs. However, as it was published in 2021, it only includes three early studies on COVID-19 measures (24). The other is a rapid review focused on COVID-19 mitigation measures that again only includes MGs from the early stages of the pandemic and not the later learnings in MG planning that evolved during the pandemic (25). Currently, no systematic review has been conducted to understand the effectiveness of such interventions specifically in sporting MGs, despite these events providing unique considerations as they often include two distinct cohorts of participants, athletes/officials and spectators, with different risk factors and minimal interactions.

This systematic review aims to address this gap and incorporate the additional learnings from COVID-19 by addressing the following research question: what type of public health interventions are implemented to mitigate respiratory infectious disease spread at sporting MGs?; how effective were they in preventing respiratory infections during the event?; and how feasible and acceptable were the interventions to implement?

## Methods

A systematic review was conducted following PRISMA guidelines. The full protocol is registered with PROSPERO (https://www.crd.york.ac.uk/prospero/display_record.php?RecordID=433619).

### Inclusion criteria, search strategy and screening

Inclusion criteria were identified aligning with the PICOS (population; intervention, comparison, outcome, study type) model:

Population: sporting MGs participants (athletes, coaches, referees, team staff, event staff, volunteers, spectators and the host community's general population).
Intervention: public health measures implemented to reduce the risk of respiratory infections.
Comparator: none; most studies are expected to be observational.
Outcomes: prevention of infections from respiratory pathogens during the event; empirical data or author narratives describing the feasibility and acceptability of implementing public health interventions during an MG.

Exclusion criteria: publications prior to 2,000, predictive modeling studies, commentaries, editorials, literature reviews, pre-prints and studies that did not retrospectively discuss the results of official sporting events or whose primary focus does not address the research questions.

The search strategy incorporated three concepts: MGs, infectious diseases and sports. A detailed search strategy is included in Supplemental File A. From June 2023, eight peer-reviewed literature [Medline; EMBASE; Cochrane Library; Scopus; Web of Science; Global Health; Epistemonikos; Global Index Medicus] and four gray-literature [WHO Library; WHO IRIS; IOC; FIFA] databases were searched. References from included studies were reviewed for additional articles to incorporate in the systematic review. In July 2025, this screening process was rerun to identify additional studies that had been published in the interim.

All articles were uploaded to Covidence (Veritas Health Innovation) (26) and duplicates removed. Each study underwent a title/abstract screening. Those that were considered potentially relevant underwent a full text screening. Two reviewers (LM and AKM) independently assessed each article during the screening processes for inclusion. A third author (CB) resolved any outstanding disagreements between these two reviewers.

### Data extraction

For each included study, one reviewer conducted the data extraction manually inputting relevant details into a structured data extraction table while a second reviewed this for completeness. Any discrepancies were resolved through discussion. The Joanna Briggs Institute (JBI) format for systematic reviews of effectiveness and of qualitative evidence was adapted for data extraction. The structured table included: article citation; title; authors; date published; data type; study type; population; outcome; host country or countries; MG under study; date MG occurred; research methods; public-health interventions, evidence about effectiveness of interventions; evidence about feasibility of interventions; evidence about acceptability of interventions; and applicability.

### Quality assessment

Quality assessment was conducted on all included studies independently by the two reviewers with discrepancies resolved through discussion. As all studies were quantitative, the ROBINS-I tool was used (27). Studies received an overall risk of bias score of “low-risk”, “moderate-risk”, “serious-risk”, “critical-risk”, or “no information” (28). Reports were not excluded based on the quality assessment. However, articles receiving an overall quality assessment score of “low-risk” or “moderate-risk” received greater weight during data synthesis.

### Data synthesis

Due to the heterogeneity of data, a statistical meta-analysis was not possible. Data were synthesized narratively across three categories: intervention descriptions; effectiveness of protocols; feasibility and acceptability of protocols.

Narrative descriptions of interventions were coded in NVivo (Version 14.23.4). Codes to describe the interventions were deductively developed, adapted from a framework of measures mitigating COVID-19 spread and a review of the data extraction tables. Codes were then inductively refined into several recurrent codes across studies (29) and used to develop a taxonomy of interventions to allow for cross-study comparison of intervention packages and connect to data describing the outcomes (Supplemental File B).

Quantitative data on infection rates and testing outcomes were synthesized into overarching categorical codes in NVivo. Codes included: cases per x population; percent positive; total number of cases reported; total number of tests conducted; percent growth; simple moving average of cases (Supplemental File B). A summary table was then developed that mapped interventions and metrics to each study with details on the approaches used to examine data and outcomes. Based on this narrative synthesis of effectiveness, each study was assessed against a pre-developed framework of effectiveness criteria to determine whether the MG under study mitigated respiratory disease spread (Table 1). The framework of effectiveness criteria was developed by the study authors for this review, adapting research they utilized on risk factor and epidemiological considerations in the development of the WHO sporting MG COVID-19 risk assessment tool and the narrative findings available from the data extraction of all included studies (30, 31). This framework was applied to each study to contextualize and provide insights into the potential effectiveness of the intervention packages implemented at MGs. This framework was designed to assess associations between intervention packages and reported disease outcomes for each MG, therefore enabling an evaluation of plausible effectiveness rather than determine causal effectiveness.

**Table 1:** Framework of effectiveness criteria.

Effectiveness classification	Criteria
Effective	Low reported cases (< 5% of participants for cases; < 1% of tests; < 10% for surveillance alerts) Low positivity rate (< 1%) No or limited intra-event transmission No major outbreaks linked to event No spillover to community High testing intensity Functioning surveillance system Rapid detection and response
Mixture (due to multiple events)	Conflicting findings on cases Some or differing reports of transmission and community impacts due to multiple events being examined Some intra-event transmission (transmission in some contexts, but not all)
Indeterminate	Insufficient detail on transmission patterns Case reports or community impacts with no comparators Unclear links between cases in event and community Small clusters but no wider spread
Not Effective	High reported cases High positivity rate presence of intra-event transmission; outbreaks Linked to event Community transmission increases linked to event

A selection of criteria was examined to determine the effectiveness of described interventions implemented at MGs. Not all criteria need to be present for determining the effectiveness classification.

Feasibility and acceptability outcomes were assessed narratively through author reported descriptions and coded in NVivo. No study used formal qualitative methods; coding for feasibility and acceptability was minimal (Supplemental File B).

Our research and author team included four women and three men, senior and less-experienced researchers with different disciplines. While the search was conducted in English, this review included all articles, regardless of the language they were published in, provided they were included in the first round of the systematic search. The articles in this review evaluated numerous events conducted in several host countries around the world and with geographically diverse participants. In discussing the generalizability of the results and limitations, we acknowledge that findings are skewed for events with more resources and international reach.

## Results

### Included study reports

**Figure 1:**
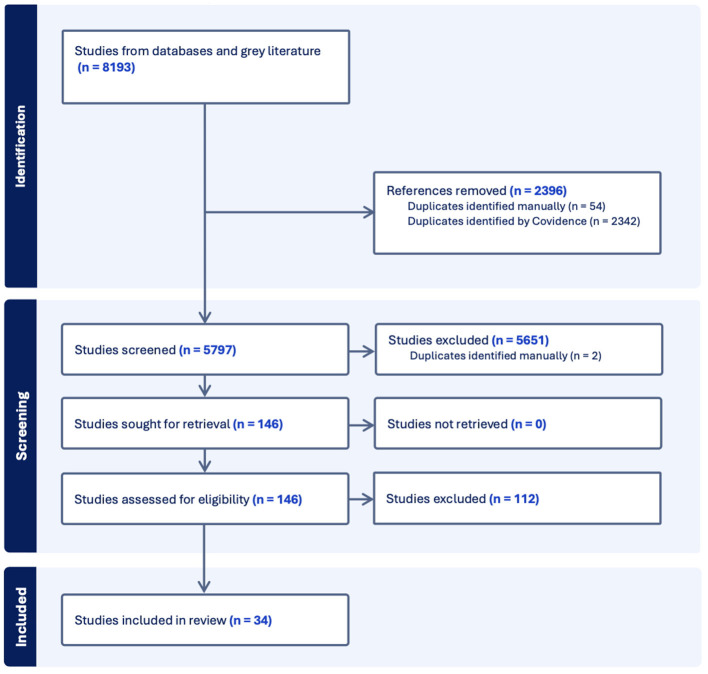
PRISMA flow diagram of article selection.

From the search strategy, 5,797 unique references were identified and screened. After the title and abstract screening, 146 study reports were retrieved for full text screening. A further 112 were removed, leaving 34 studies included in this review (Figure 1). All studies were observational and most (*n* = 33) were identified from peer-reviewed literature with the remaining being a gray-literature report (32). All studies included quantitative data on intervention effectiveness. Seven studies included author descriptions on feasibility or acceptability of interventions (33–39).

Several studies were closely discussed for potential inclusion but ultimately excluded. One did not focus solely on “official” sporting MGs but rather sports team interactions including training, theoretical events or a school sports festival (40, 41). For the scope of this research, official sporting MGs were defined as professional sports games, collegiate championships, or major national and international competitions. Other articles discussed measures in place to limit transmission of infectious diseases collectively but did not individually examine respiratory diseases (42–44).

### Study characteristics

Supplemental File C outlines the characteristics of included articles. Thirty-seven unique sporting MGs encompassing eight individual sports or a mixture of sports were represented. The most common MGs were the Olympic Games (*n* = 10) (32, 35, 37, 45–51) and the 2021 Union of European Football Association (UEFA) European Football Championship (EURO 2020) (*n* = 4) (33, 34, 52, 53). Two articles reviewed a combination of different sporting MGs, (54, 55) one of which also included EURO 2020 (55).

The most recent MGs occurred in February 2022 and included the Africa Cup of Nations 2022 (AFCON), (39, 56) Beijing 2022 Olympic Games (37, 46, 48, 51) and golf competitions (the Dimension Data Pro Am and Bain's Whisky Cape Town Open) (38). The earliest MG was the Athens 2004 Olympic Games (32). Most articles (*n* = 25) discussed MGs hosted during the COVID-19 pandemic (defined from 1 January 2020 to 5 May 2023, when WHO ended the Public Health Emergency of International Concern (PHEIC) declaration) (57) and focused solely on COVID-19 (*n* = 23). One article focused on influenza (*n* = 1) (36) while the remainder discussed a combination of respiratory diseases (*n* = 5) (49, 58–61) or respiratory diseases broadly (*n* = 5) (32, 47, 48, 54, 62).

Most studies described events with international reach, with participants attending from across the world (*n* = 17) (32, 35, 37, 38, 45–51, 55, 59, 60, 63–65). Ten articles discussed MGs with regional reach (having participants from within the region) (33, 34, 36, 39, 52, 53, 56, 58, 62, 66) and the remainder examined national MGs (participants are from within the host country) (54, 61, 67–71). Articles also covered different populations such as: athletes, team officials and event staff (*n* = 9) (36, 50, 59, 63–65, 69–71); spectators only (*n* = 3) (33, 39, 58); local host population (*n* = 8) (34, 45, 48, 49, 53, 60, 61, 68); or a combination of these with (*n* = 10) (32, 35, 38, 46, 47, 51, 52, 54, 56, 62) and without (*n* = 4) (37, 55, 66, 67) the local host population.

### Quality assessment of articles

The ROBINS-I template was used to assess the quality of quantitative methods described in each article. Findings are available in Supplemental File D. As all reports were observational with no comparators, there was considerable potential for confounding, selection bias, missing data bias and reporting bias. No articles received a low-risk score and few received a moderate-risk score (*n* = 7) (37, 45, 46, 48, 50, 65, 66). However, poor quality assessment scores were expected due to the nature of how MGs are often evaluated.

### Intervention descriptions

Table 2 describes public health measures implemented for the MG (s) assessed in each article. Interventions characterized as personal hygiene practices included hand washing and sanitizers. Enhanced venue hygiene practices included increased disinfection procedures, improved ventilation, optimizing use of outdoors, regular sanitizing of shared equipment and promoting single use of equipment.

**Table 2:** Public health measures implemented for sporting mass gatherings effectiveness results.

Reference	Sporting mass gathering under study	Public health measures implemented
Al Musleh et al. (66)	Asian Football Confederations League	Bubble; Country entry or exit testing; Enhanced hygiene practices–personal; Enhanced hygiene practices–venue; Isolation and quarantine measures; Limited capacity or no spectators; Mandatory vaccination; Mask-wearing; Minimizing physical interactions or physical distancing; Other; Routine testing; Temperature screening
Al-Thani (58)	FIFA Arab Cup	Mandatory vaccination; Mask-wearing; Other
Ayala et al. (54)	Superbowl	Enhanced surveillance
Beebeejaun et al. (33)	EURO 2020	Country entry or exit testing; Enhanced surveillance; Isolation and quarantine measures; Limited capacity or no spectators; Mandatory vaccination; Mask-wearing; Minimizing physical interactions or physical distancing; Routine testing
Berland et al.	Africa Cup of Nations (AFCON)	Country entry or exit testing; Enhanced hygiene practices–personal; Enhanced hygiene practices–venue; Enhanced surveillance; Isolation and quarantine measures; Mask-wearing; Minimizing physical interactions or physical distancing; Other; Regular health survey checks or health monitoring; Routine testing
Chowdhury et al. (45, 56)	Olympic Games (Tokyo 2020)	Bubble; Country entry or exit testing; Enhanced hygiene practices–personal; Enhanced hygiene practices–venue; Isolation and quarantine measures; Limited capacity or no spectators; Mask-wearing; Minimizing physical interactions or physical distancing; Routine testing; Temperature screening
Cuschieri et al. (34)	EURO 2020	Limited capacity or no spectators; Mask-wearing; Minimizing physical interactions or physical distancing; Routine testing; Temperature screening
De Polo et al. (63)	Cortina 2021 Alpine World Ski Championships	Bubble; Enhanced hygiene practices–personal; Enhanced hygiene practices–venue. Isolation and quarantine measures; Limited capacity or no spectators; Mask-wearing; Minimizing physical interactions or physical distancing; Regular health survey checks or health monitoring; Routine testing; Temperature screening
Dergaa et al. (35)	Olympic Games (Tokyo 2020)	Bubble; Country entry or exit testing; Isolation and quarantine measures; Limited capacity or no spectators; Mask-wearing; Minimizing physical interactions or physical distancing; Routine testing; Temperature screening
Dixon et al. (67)	NCAA Men's Basketball Tournament	Bubble; Enhanced hygiene practices–venue; Isolation and quarantine measures; Mask-wearing; Minimizing physical interactions or physical distancing; Routine testing
Fulop et al. (64)	International Swimming League 2020 Event	Bubble; Isolation and quarantine measures; Limited capacity or no spectators; Mask-wearing; Minimizing physical interactions or physical distancing; Other; Regular health survey checks or health monitoring; Routine testing
Haddad et al. (59)	6th Francophone Games	Enhanced hygiene practices–personal; Enhanced surveillance; Isolation and quarantine measures; Other
Heese et al. (52)	EURO 2020	Enhances surveillance; Limited capacity or no spectators; Mask-wearing; Other; Routine testing
Huo et al. (46)	Olympic Games (Beijing 2022)	Bubble; Country entry or exit testing; Enhanced hygiene practices–personal; Enhanced hygiene practices–venue; Isolation and quarantine measures; Mandatory vaccination; Mask-wearing; Minimizing physical interactions or physical distancing; Regular health survey checks or health monitoring; Routine testing
Kurland et al. (68)	National Football League (NFL)	Enhanced hygiene practices–venue; Limited capacity or no spectators; Minimizing physical interactions or physical distancing; Other; Routine testing
Lim et al. (36)	Asian Youth Games Singapore 2009	Isolation and quarantine measures; Mask-wearing; Other; Regular health survey checks or health monitoring; Temperature screening
McCloskey et al. (47)	Olympic Games (London 2012)	Enhanced surveillance; Other
McCloskey et al. (37)	Olympic Games (Tokyo 2020 and Beijing 2022)	Bubble; Enhanced hygiene practices–venue; Enhanced surveillance; Isolation and quarantine measures; Limited capacity or no spectators; Mask-wearing; Minimizing physical interactions or physical distancing; Other; Routine testing
Mikailova et al. (60)	FIFA World Cup	Enhanced hygiene practices–venue; Enhanced surveillance; Isolation and quarantine measures; Other; Temperature screening
Morath et al. (69)	Germany Volleyball Bundesliga 2020 Season	Isolation and quarantine measures; Mask-wearing; Minimizing physical interactions or physical distancing; Other; Routine testing
Murray et al. (70)	2020 MLB Season	Enhanced hygiene practices–venue; Isolation and quarantine measures; Limited capacity or no spectators; Mask-wearing; Minimizing physical interactions or physical distancing; Other; Regular health survey checks or health monitoring; Routine testing; Temperature screening
Nishino et al. (65)	Volleyball Nations League	Bubble; Country entry or exit testing; Enhanced hygiene practices–personal; Enhanced hygiene practices–venue; Mask-wearing; Minimizing physical interactions or physical distancing; Regular health survey checks or health monitoring; Routine testing
Pang et al. (48)	Olympic Games (Beijing 2008)	Enhanced surveillance
Pauser et al. (71)	2nd Division Professional Basketball League	Limited capacity or no spectators; Minimizing physical interactions or physical distancing; Other; Regular health survey checks or health monitoring; Temperature screening
Riccardo et al. (53)	EURO 2020	Enhanced surveillance; Limited capacity or no spectators; Mask-wearing; Minimizing physical interactions or physical distancing; Other; Routine testing
Robinson et al. (38)	Golf Competitions (Dimension Data Pro Am and Bain's Whisky Cape Town Open)	Enhanced hygiene practices–personal; Enhanced hygiene practices–venue; Isolation and quarantine measures; Mandatory vaccination; Mask-wearing; Minimizing physical interactions or physical distancing; Other; Regular health survey checks or health monitoring; Routine testing
Shimatani et al. (61)	68th National Sports Festival	Enhanced surveillance
Smith et al. (55)	Multiple Events	Limited capacity or no spectators; Other
Sugishita et al. (49)	Olympic Games (Tokyo 2020)	Enhanced surveillance; Limited capacity or no spectators
Tchounga et al. (39)	Africa Cup of Nations (AFCON)	Country entry or exit testing; Enhanced hygiene practices–personal; Isolation and quarantine measures; Mask-wearing; Minimizing physical interactions or physical distancing; Other; Routine testing
Tsouros et al. (32)	Olympic Games (Athens 2004)	Enhanced surveillance; Other
Urashima et al. (50)	Olympic Games (Tokyo 2020)	Bubble; Country entry or exit testing; Enhanced hygiene practices–personal; Enhanced hygiene practices–venue; Limited capacity or no spectators; Minimizing physical interactions or physical distancing; Other; Routine testing
White et al. (62)	8th Micronesian Games	Enhanced surveillance; Other
Xiong et al. (51)	Olympic Games (Beijing 2022)	Bubble; Enhanced hygiene practices–personal; Isolation and quarantine measures; Mandatory vaccination; Mask-wearing; Minimizing physical interactions or physical distancing; Other; Regular health survey checks or health monitoring; Routine testing

The most common interventions were physical distancing, routine testing, masking (all *n* = 21) and a general category of “other” (*n* = 22). For physical distancing, efforts included providing distinct zones and dedicated transportation to limit interactions. Regarding routine testing, five of these articles described different testing schedules for cohorts (for example athletes were tested more regularly than staff) (37, 45, 63, 67, 70). The “other” category included miscellaneous planning interventions and recommendations, such as: multisectoral task forces to review safety protocols and potential positive cases during an event; conducting regular risk assessments; stockpiling essential medications and personal-protective equipment (e.g. masks); travel advice; and entry requirements that offered options (proof of vaccination, a recent negative test or a recent infection) rather than mandating interventions.

An intervention that was used exclusively during COVID-19 was the bubble approach (*n* = 11) (35, 37, 45, 46, 50, 51, 63–67). The bubble approach is a containment strategy that reduces interaction between groups such as event participants and the local population to minimize disease spread and sometimes incorporate enhanced testing strategies or infection control measures (72). Five studies specifically discussed using multiple tiers within the bubble; not only were MG participants separated from the local host population, but their interactions with other MG participants were restricted (45, 50, 63, 64, 67). For example, athletes and coaches may be within one tier while staff and media were a separate tier, allowing for different interventions or intervention intensities to be implemented. This was often the case for testing cadence where high priority tiers such as athletes were tested more frequently that others (37, 45, 50).

Almost all articles described a multi-layered intervention package often reporting 5–10 interventions (Table 2). Bubble approaches were often implemented with routine testing (*n* = 11), (35, 37, 45, 46, 50, 51, 63–67) country entry screening (*n* = 6), (35, 45, 46, 50, 65, 66) masking (*n* = 10), (35, 37, 45, 46, 51, 63–67) physical distancing (*n* = 11) (35, 37, 45, 46, 50, 51, 63–67) and isolation and quarantine (*n* = 9) (35, 37, 45, 46, 51, 63, 64, 66, 67). Routine testing was often paired with isolation and quarantine measures (*n* = 16) (33, 35, 37–39, 45, 46, 51, 56, 60, 63, 64, 66, 67, 69, 70). Enhanced venue and personal hygiene measures often occurred simultaneously (*n* = 8) (38, 45, 46, 50, 56, 63, 65, 66). Three articles addressed only one intervention (enhanced surveillance) with no information on whether other interventions were implemented (48, 54, 61).

### Effectiveness of interventions

The most common outcomes were reported as total number of cases (*n* = 29), the percent positive or positivity rates (*n* = 12), (35, 37, 39, 46, 56, 58, 63, 64, 66, 67, 70, 71) total number of tests (*n* = 11), (35, 37, 39, 46, 50, 56, 63–67) cases per x population rate or incident rates (*n* = 8), (34, 38, 45, 52, 58, 63, 66, 67) and percent growth or percent change (*n* = 4) (34, 45, 56, 71) (Supplemental File E). Most studies used observational data with time-trend analyses (*n* = 15) (34, 35, 37, 45, 51–53, 56, 58, 62, 63, 66, 68–70) or descriptive analysis such as reporting on the distribution of cases across disease, events, geographical regions or subgroups (*n* = 31). A few studies used comparative epidemiology (*n* = 11), either between geographic regions, (34, 45, 53, 56, 63, 67, 68) MG cohort and general population, (38, 46, 51, 56, 64) or pre- and post-MG (35, 67). As there was no comparator, these analytical methods and outcomes were applied to an effective criteria framework to determine whether intervention packages were effective at mitigating respiratory disease spread (Table 3; Supplemental File F).

**Table 3:** Effectiveness of protocols to mitigate respiratory disease spread by MG.

Reference	Sporting mass gathering under study	Effectiveness determination
Al Musleh et al. (66)	Asian Football Confederations League	Effective
Al-Thani (58)	FIFA Arab Cup	Effective
Ayala et al. (54)	Superbowl	Indeterminate
Beebeejaun et al. (33)	EURO 2020	Mixture (due to multiple events)
Berland et al.	Africa Cup of Nations (AFCON)	Effective
Chowdhury et al. (45, 56)	Olympic Games (Tokyo 2020)	Indeterminate
Cuschieri et al. (34)	EURO 2020	Non effective
De Polo et al. (63)	Cortina 2021 Alpine World Ski Championships	Effective
Dergaa et al. (35)	Olympic Games (Tokyo 2020)	Effective
Dixon et al. (67)	NCAA Men's Basketball Tournament	Effective
Fulop et al. (64)	International Swimming League 2020 Event	Effective
Haddad et al. (59)	6th Francophone Games	Effective
Heese et al. (52)	EURO 2020	Effective
Huo et al. (46)	Olympic Games (Beijing 2022)	Effective
Kurland et al. (68)	National Football League (NFL)	Non effective
Lim et al. (36)	Asian Youth Games Singapore 2009	Effective
McCloskey et al. (47)	Olympic Games (London 2012)	Effective
McCloskey et al. (37)	Olympic Games (Tokyo 2020 and Beijing 2022)	Effective
Mikailova et al. (60)	FIFA World Cup	Indeterminate
Morath et al. (69)	Germany Volleyball Bundesliga 2020 Season	Indeterminate
Murray et al. (70)	2020 MLB Season	Indeterminate
Nishino et al. (65)	Volleyball Nations League	Effective
Pang et al. (48)	Olympic Games (Beijing 2008)	Effective
Pauser et al. (71)	2nd Division Professional Basketball League	Non effective
Riccardo et al. (53)	EURO 2020	Indeterminate
Robinson et al. (38)	Golf Competitions (Dimension Data Pro Am and Bain's Whisky Cape Town Open)	Effective
Shimatani et al. (61)	68th National Sports Festival	Effective
Smith et al. (55)	Multiple Events	Mixture (due to multiple events)
Sugishita et al. (49)	Olympic Games (Tokyo 2020)	Effective
Tchounga et al. (39)	Africa Cup of Nations (AFCON)	Effective
Tsouros et al. (32)	Olympic Games (Athens 2004)	Effective
Urashima et al. (50)	Olympic Games (Tokyo 2020)	Effective
White et al. (62)	8th Micronesian Games	Effective
Xiong et al. (51)	Olympic Games (Beijing 2022)	Effective

Based on this framework, 23 articles were associated with effective intervention packages. Almost all (*n* = 6) articles that received a moderate-risk quality assessment rating were included (37, 46, 48, 50, 65, 66). Three articles were associated with non-effective intervention packages, (34, 68, 71) two of which had a critical-risk quality assessment score, one that did not provide the number of individuals in the study and the other that contained 61 individuals (34, 71). The remaining article reviewed 269 NFL games and had a serious-risk score (68). Six articles were associated with indeterminate intervention packages, (45, 53, 54, 60, 69, 70) including the final article with a moderate-risk quality assessment score that assessed 42 individuals (45). Two articles were classified as describing intervention packages for multiple MGs where interventions were likely effective in some settings but not all, with critical-risk score that reviewed 51 football matches across 11 host countries and serious-risk quality assessment score that reviewed eight MGs or groups of MGs (33, 55). Most events associated with effective outcomes, particularly the 6 reports with moderate-risk quality assessment scores, reported low number of cases, high testing volumes and no spillover to the host community. Examples included the Asian Football Confederations League and Olympic Games (32, 35, 37, 39, 46–51, 56, 63, 66, 67). MGs classified with non-effective interventions included the National Football League and the 2^nd^ Division Professional Basketball League, both of which reported high case counts linked to the MGs, community spillover or intra-event transmission (68, 71). However, such articles did not address confounders including COVID-19 prevalence within the community and the impact of the national restrictions in place. Studies describing EURO 2020 were classified across the effectiveness criteria framework, depending on the match and population. For example, one study reported German matches did not have a significant impact on the COVID-19 outbreak within the country with a low number of cases identified from spectators and limited intra-event transmission. However, the study had multiple biases leading to a critical-risk quality assessment score (52). Another study outlined interventions for stadium attendees during Italian matches and reported low number of cases and minimal event transmission. However, despite noting the several unofficial watch parties that also occurred with minimal interventions and resulted in several outbreaks, this article did not account for a number of other confounders, resulting in a critical-risk score (33). Other studies reviewed EURO 2020 impact to the general population and reported sharp increases in COVID-19 across host cities and countries, but did not necessarily account for the ongoing pandemic phases and impact of the lifting of local restrictions (34, 55). Reviewing all articles against the effectiveness criteria framework showed that international MGs with larger and multi-layered intervention packages were associated with more effective outcomes. This was likely due to such events having more resources and dedicated planning teams.

Table 4 compares the interventions listed across articles and the respective effectiveness classification associations. Patterns showed MGs that implemented an intervention package including bubble approaches, isolation and quarantine measures, mask-wearing, routine testing, limiting physical distancing, and country entry testing were commonly associated with effective outcomes. Five of the seven articles that scored a moderate-risk on the quality assessment and considered effective against the framework, implemented bubble approaches, routine testing and country entry/exit testing (37, 46, 50, 65, 66). In particular, during country infection peaks, such as those highlighted in Tokyo 2020 or Beijing 2022 articles, bubble approaches and routine testing were vital to the quick identification of cases and reducing risk of further spread (8, 35, 45, 46, 49–51). Temperature screening, limiting capacity of spectators, promoting enhanced hygiene practices and enhanced surveillance systems had mixed results, especially if they were not incorporated with several other interventions, such as testing.

**Table 4:** Number of articles with various public health measures by reported effectiveness of interventions for MG.

Public health measures	Effective	Non effective	Indeterminate	Mixture (due to multiple events)
Bubble	10	0	1	0
Country entry or exit testing	7	0	1	1
Enhance hygiene practices–personal	10	0	1	0
Enhanced hygiene practices–venue	7	0	3	0
Enhanced surveillance	10	0	3	1
Enhanced ventilation	4	1	0	0
Isolation and quarantine measures	13	0	4	1
Limited capacity or no spectators	8	3	3	2
Mandatory vaccination	5	0	0	1
Mask-Wearing	15	1	4	1
Minimizing Physical Interactions or physical distancing	13	3	4	1
Other	15	2	4	1
Regular health survey checks or health monitoring	8	1	1	0
Routine Testing	14	2	4	1
Temperature screening	4	2	3	0

### Feasibility and acceptability of interventions

Feasibility and acceptability of interventions were sparsely mentioned across included studies (33–39). In addition such findings were minimal author narratives or reports with no analysis or evidence to further support the exploratory findings. Two articles incorporated anecdotal feasibility findings with mixed results (38, 39). One article noted that no participants reported an increased burden of work despite the additional interventions in place (38). Another article briefly reported challenges of implementing routine testing and health passes at fan zones; the high attendance caused testing scalability issues and led to increased financial pressures. Logistical challenges with managing the increased workload were also briefly reported by the author (39).

Seven articles included brief author reports describing acceptability of interventions with a mixture of articles expressing positive and negative descriptions (8, 33–36, 38, 39). Two articles assessing EURO 2020 mentioned low compliance with interventions and external pressure to increase stadium capacity size hindering physical distancing and limited spectator interventions (33, 34). Another study expressed frustrations from hotel operators with their expected responsibilities of maintaining bubble strategies and instances of noncompliance from international visitors (35). Similarly, one article reported aggressive behaviors, lobbying by external stakeholders and a decrease in expected spectators at events due to wait times for entry testing (39). Articles describing the Asian Youth Games and Tokyo 2020 and Beijing 2022 reported high levels of compliance with interventions (36, 37). The article that included positive stakeholder perceptions on the feasibility of interventions reported similar sentiments from athletes and staff (38). Similarly to patterns identified in feasibility analysis, an influencing factor for accessibility was the population under study. Implementing intervention packages on large groups such as spectators or at fan zones provided more feasibility and acceptability challenges than smaller, structured cohorts including athletes and staff.

## Discussion

### Summary of key findings

This systematic review provides the first opportunity to comprehensively compare pre-pandemic and pandemic-era planning for sporting MGs, highlighting how multilayered intervention packages can meaningfully reduce the risk of respiratory disease spread. While there was some empirical evidence prior to the pandemic on the effectiveness of interventions at MGs, the COVID-19 pandemic provided opportunities to increase understanding and improve the evidence base for MG planning. The variation in event types, geographic settings and participant profiles included in this review offer a valuable opportunity to also explore how contextual factors shape the effectiveness of public health interventions implemented at sporting MGs. This research builds off of findings from prior MG reviews that identify health threats and list mitigation measures implemented (16, 19, 21, 24). With a specific focus on sporting MGs, this review uniquely applied all studies to a framework to evaluate the effectiveness of the described intervention packages. This review also highlights how surveillance measures have increased in event planning with routine testing as a regular intervention for MGs held during the pandemic (16, 20).

Synthesized findings from this review showed events implementing layered interventions were frequently associated with effective outcomes. Reducing interaction between participants (through bubble approaches, physical distancing and limiting spectators), increasing early detection of cases (through country entry testing, routing testing, enhanced surveillance systems and health surveys) and ensuring the quick isolation and quarantine of confirmed or suspected cases were potential drivers of effective outcomes. This remains in line with broader evidence of infection prevention; evidence from influenza, SARS, MERS and COVID-19 demonstrate limiting the population density and opportunities for close contact through efforts including distancing and reducing crowd size are key measures in minimizing respiratory disease spread (73–77). Bubble approaches implemented at some MGs provide stricter contact limitations which were often associated with more effective outcomes. Early detection and isolation of cases is also well-documented in literature as a key driver for outbreak control (). MG interventions that incorporate multiple testing approaches including country entry testing and regular testing throughout the event align with the recognized best practices for outbreak control, minimizing opportunities for clusters to grow or spillover into the host population (74, 75, 78).

Contextual factors influenced the effectiveness of interventions. National policies that followed stringent protocols were able to prevent spillover or reported minimal cases in the host population linked to the event as highlighted by Tokyo 2020, Beijing 2022 and the Asia Youth Games. Conversely, events in settings will less stringent policies in place, such as the National Football League, reported multiple increases in cases and association between crowd sizes and higher post-event increases. Transmission dynamics and infection peaks within the community had the potential to impact intervention effectiveness, particularly if bubble approaches were not used and spectators allowed, such as during EURO 2020. Other factors unique to the MG (s) under study also likely influenced the effectiveness of interventions, particularly the infection transmission rates. For example, seasonality trends of certain respiratory diseases, whether the event was held in an indoor or outdoor venue, and the level of close contact the sport would require, would all impact infection opportunities and the implementation of interventions. However, the articles included in this literature review did not provide detail on these confounders to allow for a comparison across sports and MGs.

The COVID-19 pandemic saw more stringent interventions implemented at MGs focused on separating population cohorts and implementing routine testing, than seen in prior MGs. Table 5 maps the MG events, cataloged by time period (pre-pandemic, during COVID-19), that implemented various interventions. Certain interventions that are resource intensive were commonly used during the pandemic. Other interventions also saw a resurgence including enhanced venue hygiene, personal hygiene practices and masking. These measures are typically less cost prohibitive but can make significant impacts to reducing disease spread.

**Table 5:** Number of articles with various public health measures by MG event era.

Public health measures	Pre COVID	During COVID	Post COVID	Combination of event eras
Bubble	0	11	0	0
Country entry or exit testing	0	9	0	0
Enhance hygiene practices–personal	1	10	0	0
Enhanced hygiene practices–venue	1	9	0	0
Enhanced surveillance	8	6	0	0
Enhanced ventilation	0	5	0	0
Isolation and quarantine measures	3	15	0	0
Limited capacity or no spectators	0	16	0	0
Mandatory vaccination	0	6	0	0
Mask-Wearing	1	20	0	0
Minimizing Physical Interactions or physical distancing	0	21	0	0
Other	6	16	0	0
Regular health survey checks or health monitoring	1	9	0	0
Routine Testing	0	21	0	0
Temperature screening	2	7	0	0

Narrative synthesis of feasibility and acceptability identified potential factors that constrained the implementation of interventions. Feasibility concerns appeared a challenge for MGs with a high number of spectators and linked to scalability issues (39). Acceptability factors were likely influenced by the perception of increased work burden, compliance levels and stakeholder engagement. Study authors summarized that MG participants reported interventions were more acceptable in small, closed environments, such as between athletes and staff who perceived them as necessary for the competition to occur (37, 38). Compliance was described as an enabling mechanism that had the potential to contribute to limiting or increasing disease spread. In highly regulated environments such as the Olympic Games, study authors often described high compliance with interventions and an MG that was reported as effective in mitigate disease spread. In events with low enforcement and public opposition such as during EURO 2020 interventions had limited effect.

### Limitations

There are several limitations of this systematic review. Despite an exhaustive search of databases and a wide time range, relevant study reports were likely missed, especially unpublished evaluations and internal reports (79, 80). There is a disproportionate focus on major MGs, while smaller MGs, particularly from low- and middle-income countries, were underrepresented. Larger MGs often require greater levels of planning and resources, making them not directly comparable to other sporting MGs. Due to the number of studies published on Tokyo 2020 and Beijing 2022, this systematic review incorporated several findings from MGs that occurred in Asian countries where cases of COVID-19 were often lower than the rest of the world, there was high compliance of national restrictions and national policies were more stringent. Similarly, most studies focused solely on COVID-19 mitigation. The limited disease and geographical diversity may skew findings and limit generalizability.

Due to the heterogeneity of interventions and study methods, a meta-analysis was not possible. Events varied considerably in scale, population under study, epidemiological context and analytical approaches used to assess outcomes. Such differences limit the comparability of findings across studies as outcomes and findings from one study may not be transferable to another MG. Therefore, the synthesis provided in this review relied on identifying recurring patterns and associations. The heterogeneity of this review further highlights the need for context-sensitive interpretation and consideration.

Narrative synthesis was conducted for this systematic literature review which is subject to bias. While articles providing higher quality methods were given greater weight, following the quality assessment, no article scored above a moderate-risk score. The effectiveness criteria framework that each study was compared against to identify interventions associated with effective outcomes has not undergone independent, external validation. Although it was informed by established epidemiological principles and prior sporting MG risk assessment research, its development and use within this review may introduce bias. There is potential for circular reasoning if intervention components aligned with best practices are also used to interpret reported outcomes as indicators of potential effectiveness. Future research should seek to externally test and validate the framework across MG contexts. In addition, while this literature review also aimed to determine feasibility and acceptability of interventions, few studies incorporated such findings. Those that did provided minimal narrative descriptions and no qualitative findings, significantly limiting the depth of analysis that could be conducted. Therefore, these findings should be considered exploratory and further, dedicated studies need to be completed to appropriately assess feasibility and acceptability considerations.

Within the studies themselves, all were observational and none employed experimental or quasi-experimental designs. Articles described to differing levels of details confounding factors and contexts in terms of infection prevalence and pandemic phase as well as in local population and visiting population characteristics. Many additional confounders were often not discussed or differentiated in articles. For example, the impact of seasonality on respiratory infections was not considered. Articles did not provide the ability to compare infection transmission for indoor vs. outdoor events or between sports that required close contact vs. single individual sports. Few articles examined a single intervention and those that did focused on enhanced surveillance measures but did not address what other public health measures may have been in place. As most studies focused on interventions that involved multiple components, it was impossible to make an assessment on the degree of effectiveness for individual measures. In addition, there was inconsistent reporting of intervention details including length of implementation and degree of enforcement which limited the inferences made on intervention effectiveness.

### Implications for policy and research

This systematic review underscores the importance of multilayered, context-specific intervention packages to reduce the risk of respiratory disease spread. While it may be difficult to draw conclusions on which interventions are most effective for MGs, this review highlights the challenges of generalizability due to varied events, settings, pathogens and implementation quality and provides evidence that a package of interventions likely impacts effort to mitigate disease spread. This review also aligns with existing evidence on infection prevention and control measures. Using multilayered intervention packages matches the established “Swiss Cheese Model” that is well recognized in infection control and promotes the use of multiple interventions to collective reduce disease transmission risk (74, 75, 78). This review has provided MG event organizers with different approaches, using a variety of interventions, for the planning and hosting of their upcoming events. Future MGs should utilize a risk-based, layered approach to designing safety protocols and choose interventions tailored to disease characteristics, nature of the threat, size and nature of the MG and host country health capacity.

From a policy perspective, findings from this review emphasize the need for early MG planning and coordination, similar to how the broader evidence shows strong surveillance, early detection and isolation are key measures to effective disease control (78, 81–83). Intensive intervention packages, such as routine testing, enhanced surveillance and country entry or exit testing will need country support or approval. The cost-prohibitive nature of certain interventions, such as bubble approaches and routine testing, will likely limit their implementation to settings where the risk of disease spread is considerable, such as during ongoing outbreaks. It should also be noted that some interventions may only be impactful in specific settings and with specific pathogens. For example, to mitigate influenza spread, fomite-focused interventions (regular cleaning of surfaces and equipment) could be useful but may be less impactful for diseases that are spread primarily through inhalation and aerosols. Event organizers should review the epidemiology and transmission dynamics of their target diseases to ensure the interventions in place are applicable. MGs of all sizes and context should make efforts to actively share learnings and experiences to provide further evaluations of effective approaches across settings and diseases. Regardless of the interventions in place, clear communication strategies for organizers, national authorities and event participants will improve feasibility and acceptability challenges.

Evidence gaps in the literature include qualitative studies that showcase participant perspectives and implementation experiences. Very few articles were screened that incorporated any data on how interventions were implemented, enforced, sustained or perceived. Those articles that did incorporate such considerations, only provide minimal insights as author narratives and exploratory findings. In particular, feasibility and acceptability of interventions remain areas where further research is greatly warranted to map the burden of implementing intervention packages and the overall levels of acceptability. This information will update current understanding of recognized best practices for MG planning, providing the nuance and contextual insight into why interventions may work in some settings but not others. Such future research should be considered a priority for MG planning. Contributions to research should also examine MGs post COVID-19. Such events may incorporate learnings from the pandemic and whether MG planning or intervention packages have evolved. Evaluation studies should also be conducted, with a particular focus on cost-effectiveness and scalability for low- and middle-income contexts.

## Data Availability

The original contributions presented in the study are included in the article/Supplementary material, further inquiries can be directed to the corresponding author.
