# Supplementary material for: Effectiveness of public health measures and strategies to reduce risk of spread of respiratory pathogens at sporting mass gatherings: systematic literature review
Source: Front Public Health. 2026 Apr 8;14:1789413. doi: 10.3389/fpubh.2026.1789413 (PMC13099540; doi:10.3389/fpubh.2026.1789413)
Supplement: Supplementary file 2 [file Data_Sheet_1.pdf]

SYSTEMATC LITERATURE REVIEW SEARCH CRITERIA AND STRATEGY

SEARCH CONCEPTS

Concept 1: Mass Gatherings	Concept 2: Infectious Diseases	Concept 3: Sports
mass gathering*	Infectious disease	Sport*
Crowding	Virus	Competition*
Gathering	Disease	Olympic*
Mass	Outbreak	World Cup*
Large	Epidemic	FIFA
Big	Pandemic	UEFA
Excessive	Influenza	Super bowl
Gather*	Flu	Test match
Event*	COVID	Play-off*
Crowd	COVID-19	Final
	Ebola	Finals
	MERS	Tournament*
	SARS	Game*
	Coronavirus	Cup
	SARS-CoV-2	
	Communicable	

DATABASES:

Peer reviewed literature databases:

- Medline
- EMBASE
- Cochrane Library
- Scopus
- Web of Science
- Global Health
- Epistemonikos
- Global Index Medicus

Grey literature databases and document libraries:

- WHO (WHO Library + IRIS)
- IOC
- FIFA

EXAMPLE SEARCH STRATEGY FOR OVID DATABASES

1	mass gathering*.mp.
2	crowding.mp.
3	gathering.mp.
4	((mass or large or big or excessive) adj5 (gather* or event* or crowd)).mp.
5	Mass Gatherings/
6	1 or 2 or 3 or 4 or 5
7	infectious disease.mp.
8	(virus* or disease* or infect* or outbreak* or epidemic* or pandemic* or influenza or flu or COVID* or "covid-19" or Ebola or MERS or SARS or coronavirus or SARS-CoV-2).mp.
9	exp disease outbreaks/ or exp epidemics/
10	exp Virus Diseases/
11	Communicable Diseases/
12	7 or 8 or 9 or 10 or 11
13	(sport* or competition* or olympic* or "world cup*" or FIFA or EUFA or "super bowl*" or "test match" or "play-off*" or final or finals or tournament* or game* or cup).mp.
14	exp Sports/
15	13 or 14
16	**6 and 12 and 15**
