# Supplementary material for: Effectiveness of public health measures and strategies to reduce risk of spread of respiratory pathogens at sporting mass gatherings: systematic literature review
Source: Front Public Health. 2026 Apr 8;14:1789413. doi: 10.3389/fpubh.2026.1789413 (PMC13099540; doi:10.3389/fpubh.2026.1789413)
Supplement: Supplementary file 3 [file Data_Sheet_2.pdf]

Supplemental File B (Table): Additional Codes, Categories and Criteria for Data Synthesis

Intervention Codes		Intervention Framework
**Deductive and inductive codes**	One bubble;<br>levels of interaction within a bubble;<br>Country entry testing;<br>Country exit testing;<br>Venue cleaning,<br>Ventilation, surveillance;<br>Isolation and quarantine;<br>Limited capacity;<br>No spectators;<br>Vaccination;<br>Masking;<br>Physical distancing;<br>Regular health surveys;<br>Symptomatic checks;<br>MG entry testing;<br>MG routine testing	Bubble<br>Country entry or exit testing<br>Enhanced hygiene practices – personal<br>Enhanced hygiene practices – venue<br>Enhanced surveillance<br>Isolation and quarantine measures<br>Limited capacity or no spectators<br>Mandatory vaccination<br>Mask-wearing<br>Minimizing physical interactions or physical distancing<br>Other<br>Regular health survey checks or health monitoring<br>Routine testing<br>Temperature screening
**Feasibility and Acceptability Codes**		
**Deductive codes**	Feasibility Findings	Acceptability Findings
**Inductive codes**	Financial constraints;<br>Logistical capacity;<br>Scalability;<br>Reports of stakeholder perceptions	Compliance with measures;<br>Voiced opposition;<br>Reports of stakeholder perceptions
**Effectiveness**	**Quantitative Metrics Categories**	**Approaches Used for Effectiveness Analysis**
	Cases per x population / incident rate<br>Percent positive / positive rate / attack rate<br>Total number or cases/incidents/events reported<br>Total number of tests conducted<br>Percent growth / percent increases / percent change<br>Simple moving average of cases/rates	Time trend for case detection;<br>Comparative epidemiology or trend analysis;<br>Effectiveness of specific interventions;<br>Distribution of cases across subgroups;<br>Testing intensity and surveillance coverage;<br>Associations between factors under study;<br>Modelled projections
**Effectiveness Criteria Framework**		
**Effective**	Low reported cases;<br>Low positivity rate;<br>No or limited intra-event transmission;<br>No major outbreaks linked to event;<br>No spillover to community;<br>High testing intensity;<br>Functioning surveillance system;<br>Rapid detection and response	
**Mixed (due to multiple events)**	Conflicting findings on cases;<br>Transmission and community impacts due to multiple events being examined;<br>Some intra-event transmission (transmission in some contexts, but not all)	
**Indeterminate**	Insufficient detail on transmission patterns;<br>Case reports or community impacts;<br>No comparators;	

	Unclear links between cases in event and community;<br>Small clusters but no wider spread
**Non effective**	High reported cases;<br>High positivity rate;<br>Presence of intra-event transmission;<br>Outbreaks linked to event;<br>Community transmission increases linked to event
