# Supplementary material for: Effectiveness of public health measures and strategies to reduce risk of spread of respiratory pathogens at sporting mass gatherings: systematic literature review
Source: Front Public Health. 2026 Apr 8;14:1789413. doi: 10.3389/fpubh.2026.1789413 (PMC13099540; doi:10.3389/fpubh.2026.1789413)
Supplement: Supplementary file 4 [file Data_Sheet_3.pdf]

Supplemental File C (Table): Characteristics of Included Articles

Article	Host Country	MG Reach	Disease	Population Under Study	Event Era or Time Period	MG Event	MG Dates	Sport	Study Type	Data Type
**Al Musleh et al_2022**[66]	Qatar	Regional	COVID-19	Combination (without local host population)	During COVID	Asian Football Confederations League	November – December 2020	Football	Observational: descriptive cohort study	Quantitative
**Al-Thani_2022**[58]	Qatar	Regional	Combination	Spectators	During COVID	FIFA Arab Cup	November – December 2021	Football	Observational: cross sectional study	Quantitative
**Ayala et al_2016**[54]	USA	Local / National	Respiratory (Unspecified or general)	Combination (with local host population)	Pre COVID	Mixture of Events<br><br>(Superbowl XLIX; Pro Bowl; NFL Experience; Superbowl Central; Fiesta Bowl; Waste Management Pheonix Open)	February 2015	American Football	Observational: narrative study	Quantitative
**Beebeejaun et al_2022**[33]	Multiple (Europe)	Regional	COVID-19	Spectators	During COVID	EURO 2020	June – July 2021	Football	Observational: retrospective study	Quantitative
**Berland et al_2024**	Cameroon	Regional	COVID-19	Combination (with local host population)	During COVID	Africa Cup of Nations (AFCON)	January – February 2022	Football	Observational: retrospective study	Quantitative
**Chowdhury et al_2023**[45,56]	Japan	International / Global	COVID-19	Local Host Population	During COVID	Olympic Games<br><br>(Tokyo 2020)	July – September 2021	Mixture of Sports	Observational: cohort study	Quantitative
**Cuschieri et al_2022**[34]	Multiple (Europe)	Regional	COVID-19	Local Host Population	During COVID	EURO 2020	June – July 2021	Football	Observational: retrospective study	Quantitative
**De Polo et al_2021**[63]	Italy	International / Global	COVID-19	Athletes, Team Officials and Event Staff	During COVID	Cortina 2021 Alpine World Ski Championships	February 2021	Skiing	Observational: narrative study	Quantitative
**Dergaa et al_2022**[35]	Japan	International / Global	COVID-19	Combination (with local host population)	During COVID	Olympic Games<br><br>(Tokyo 2020)	July – September 2021	Mixture of Sports	Observational: narrative study	Quantitative

**Dixon et al_2022**[67]	USA	Local / National	COVID-19	Combination (without local host population)	During COVID	NCAA Men's Basketball Tournament	March – April 2021	Basketball	Observational: retrospective cohort study	Quantitative
**Fulop et al_2022**[64]	Hungary	International / Global	COVID-19	Athletes, Team Officials and Event Staff	During COVID	International Swimming League 2020 Event	October 2022	Swimming	Observational: retrospective narrative study	Quantitative
**Haddad et al_2017**[59]	Lebanon	International / Global	Combination	Athletes, Team Officials and Event Staff	Pre COVID	6th Francophone Games	September – October 2009	Mixture of Sports	Observational: retrospective narrative study	Quantitative
**Heese et al_2022**[52]	Germany	Regional	COVID-19	Combination (with local host population)	During COVID	EURO 2020	June – July 2021	Football	Observational: descriptive case study	Quantitative
**Huo et al_2023**[46]	China	International / Global	COVID-19	Combination (with local host population)	During COVID	Olympic Games<br><br>(Beijing 2022)	January – February 2022	Mixture of Sports	Observational: case study	Quantitative
**Kurland et al_2022**[68]	USA	Local / National	COVID-19	Local Host Population	During COVID	National Football League (NFL)	March 2020 – March 2021	American Football	Observational: cross sectional study	Quantitative
**Lim et al_2010**[36]	Singapore	Regional	Influenza	Athletes, Team Officials and Event Staff	Pre COVID	Asian Youth Games Singapore 2009	June – July 2009	Mixture of Sports	Observational: narrative synthesis	Quantitative
**McCloskey et al_2014**[47]	UK	International / Global	Respiratory (Unspecified or general)	Combination (with local host population)	Pre COVID	Olympic Games<br><br>(London 2012)	July – September 2012	Mixture of Sports	Observational: retrospective narrative study	Quantitative
**McCloskey et al_2024**[37]	Multiple (Asia)	International / Global	COVID-19	Combination (without local host population)	During COVID	Olympic Games<br><br>(Tokyo 2020 and Beijing 2022)	July – August 2021 (Tokyo);<br>February – March 2021 (Beijing)	Mixture of Sports	Observational: retrospective narrative study	Quantitative
**Mikailova et al_2020**[60]	Russia	International / Global	Combination	Local Host Population	Pre COVID	FIFA World Cup	June – July 2018	Football	Observational: retrospective narrative study	Quantitative
**Morath et al_2022**[69]	Germany	Local / National	COVID-19	Athletes, Team Officials and Event Staff	During COVID	Germany Volleyball Bundesliga 2020 Season	September – December 2020	Volleyball	Observational: retrospective cohort study	Quantitative

**Murray et al_2020**[70]	USA	Local / National	COVID-19	Athletes, Team Officials and Event Staff	During COVID	2020 MLB Season	July – September 2020	Baseball	Observational: case study	Quantitative
**Nishino et al_2022**[65]	Italy	International / Global	COVID-19	Athletes, Team Officials and Event Staff	During COVID	Volleyball Nations League	May – June 2021	Volleyball	Observational: case study	Quantitative
**Pang et al_2017**[48]	China	International / Global	Respiratory (Unspecified or general)	Local Host Population	Pre COVID	Olympic Games<br><br>(Beijing 2008)	August 2008	Mixture of Sports	Observational: narrative case study	Quantitative
**Pauser et al_2021**[71]	Germany	Local / National	COVID-19	Athletes, Team Officials and Event Staff	During COVID	2nd Division Professional Basketball League	November 2020	Basketball	Observational: retrospective case study	Quantitative
**Riccardo et al_2022**[53]	Italy	Regional	COVID-19	Local Host Population	During COVID	EURO 2020	June – July 2021	Football	Observational: retrospective narrative study	Quantitative
**Robinson et al_2022**[38]	South Africa	International / Global	COVID-19	Combination (with local host population)	During COVID	Golf Competitions (Dimension Data Pro Am and Bain's Whisky Cape Town Open)	February 2022	Golf	Observational: prospective cohort study	Quantitative
**Shimatani et al_2015**[61]	Japan	Local / National	Combination	Local Host Population	Pre COVID	68th National Sports Festival	September – October 2013	Mixture of Sports	Observational: case study	Quantitative
**Smith et al_2022**[55]	UK	International / Global	COVID-19	Combination (without local host population)	During COVID	Multiple Events<br><br>(International cricket; EURO 2020; Royal Ascot; Download Festival; Wimbledon Tennis; Goodwood Festival; The Open Golf)	June – July 2021	Mixture of Sports	Observational: retrospective cohort study	Quantitative
**Sugishita et al_2023**[49]	Japan	International / Global	Combination	Local Host Population	During COVID	Olympic Games<br><br>(Tokyo 2020)	July – September 2021	Mixture of Sports	Observational: narrative study	Quantitative
**Tchounga et al_2025**[39]	Cameroon	Regional	COVID-19	Spectators	During COVID	Africa Cup of Nations (AFCON)	January – February 2022	Football	Observational: cross sectional study	Quantitative

**Tsouros et al_2007**[32]	Greece	International / Global	Respiratory (Unspecified or general)	Combination (with local host population)	Pre COVID	Olympic Games<br><br>(Athens 2004)	August - September 2004	Mixture of Sports	Grey Literature	Quantitative
**Urashima et al_2022**[50]	Japan	International / Global	COVID-19	Athletes, Team Officials and Event Staff	During COVID	Olympic Games<br><br>(Tokyo 2020)	July – September 2021	Mixture of Sports	Observational: prospective cohort study	Quantitative
**White et al_2018**[62]	Federated States of Micronesia	Regional	Respiratory (Unspecified or general)	Combination (with local host population)	Pre COVID	8th Micronesian Games	July 2014	Mixture of Sports	Observational: narrative case study	Quantitative
**Xiong et al_2023**[51]	China	International / Global	COVID-19	Combination (with local host population)	During COVID	Olympic Games<br><br>(Beijing 2022)	February 2022	Mixture of Sports	Observational: retrospective case study	Quantitative
