# Supplementary material for: Effectiveness of public health measures and strategies to reduce risk of spread of respiratory pathogens at sporting mass gatherings: systematic literature review
Source: Front Public Health. 2026 Apr 8;14:1789413. doi: 10.3389/fpubh.2026.1789413 (PMC13099540; doi:10.3389/fpubh.2026.1789413)
Supplement: Supplementary file 5 [file Data_Sheet_4.pdf]

Supplemental File D (Table): Quantitative Article Quality Assessment Ratings (ROBINS-I)

Key:

Low

Moderate

Serious

Critical

NI

Article	Confounding	Selection Bias	Classification of Interventions	Deviation from Intended Interventions	Missing Data	Measurement of Outcomes	Selection of Reported Results	Overall
Al Musleh et al_2022[66]	Moderate	Low	Low	Moderate	Low	Moderate	Moderate	Moderate
Al-Thani_2022[58]	Serious	Serious	Low	Low	Low	Low	Low	Serious
Ayala et al_2016[54]	Critical	Moderate	Low	Low	Low	Low	Moderate	Critical
Beebeejaun et al_2022[33]	Serious	Critical	Serious	Serious	Serious	Low	Low	Critical
Berland et al_2024	Serious	Moderate	Moderate	Serious	Serious	Low	Serious	Serious
Chowdhury et al_2023[45,56]	Moderate	Moderate	Low	Low	Low	Low	Low	Moderate
Cuschieri et al_2022[34]	Critical	Moderate	Low	Serious	Moderate	Low	Moderate	Critical
De Polo et al_2021[63]	Serious	Low	Low	Moderate	Low	Moderate	Moderate	Serious
Dergaa et al_2022[35]	Critical	Low	Low	Serious	Low	Low	Low	Critical
Dixon et al_2022[67]	Serious	Low	Low	Moderate	Low	Moderate	Moderate	Serious

Fulop et al_2022[64]	− (yellow)	− (green)	− (green)	+ (orange)	− (green)	− (green)	− (yellow)	+ (orange)
Haddad et al_2017[59]	− (yellow)	− (yellow)	− (green)	× (red)	+ (orange)	+ (orange)	− (green)	× (red)
Heese et al_2022[52]	+ (orange)	× (red)	− (yellow)	+ (orange)	× (red)	− (green)	− (yellow)	× (red)
Huo et al_2023[46]	− (yellow)	− (green)	− (green)	− (yellow)	− (green)	− (green)	− (green)	− (yellow)
Kurland et al_2022[68]	+ (orange)	− (yellow)	− (green)	? (grey)	− (yellow)	− (green)	− (green)	+ (orange)
Lim et al_2010[36]	× (red)	+ (orange)	− (green)	− (green)	− (yellow)	− (green)	− (green)	× (red)
McCloskey et al_2014[47]	+ (orange)	− (yellow)	− (green)	− (green)	− (yellow)	− (green)	− (yellow)	+ (orange)
McCloskey et al_2024[37]	− (yellow)	− (green)	− (yellow)	− (green)	− (green)	− (green)	− (green)	− (yellow)
Mikailova et al_2020[60]	× (red)	− (yellow)	+ (orange)	? (grey)	? (grey)	− (yellow)	− (yellow)	× (red)
Morath et al_2022[69]	× (red)	+ (orange)	+ (orange)	+ (orange)	? (grey)	− (green)	− (green)	× (red)
Murray et al_2020[70]	+ (orange)	− (green)	− (green)	− (green)	− (green)	+ (orange)	− (yellow)	+ (orange)
Nishino et al_2022[65]	− (yellow)	− (green)	− (green)	− (yellow)	− (yellow)	− (green)	− (green)	− (yellow)
Pang et al_2017[48]	− (yellow)	− (green)	− (green)	− (green)	− (green)	− (green)	− (green)	− (yellow)
Pauser et al_2021[71]	× (red)	− (yellow)	+ (orange)	? (grey)	− (yellow)	+ (orange)	− (green)	× (red)
Riccardo et al_2022[53]	+ (orange)	− (yellow)	+ (orange)	× (red)	+ (orange)	− (yellow)	+ (orange)	× (red)
Robinson et al_2022[38]	+ (orange)	− (green)	− (green)	? (grey)	× (red)	+ (orange)	− (yellow)	× (red)
Shimatani et al_2015[61]	+ (orange)	− (yellow)	− (green)	− (green)	− (yellow)	− (green)	− (yellow)	+ (orange)
Smith et al_2022[55]	− (yellow)	+ (orange)	− (yellow)	− (yellow)	− (yellow)	− (green)	− (green)	+ (orange)
Sugishita et al_2023[49]	+ (orange)	− (green)	+ (orange)	− (green)	− (yellow)	− (yellow)	− (yellow)	+ (orange)
Tchounga et al_2025[39]	+ (orange)	− (yellow)	− (yellow)	+ (orange)	− (yellow)	− (green)	− (yellow)	+ (orange)

Tsouros et al_2007[32]	+	−	−	−	−	−	−	+
Urashima et al_2022[50]	−	−	−	−	−	−	−	−
White et al_2018[62]	×	−	−	−	−	−	−	×
Xiong et al_2023[51]	−	+	−	−	−	−	−	+
