# Supplementary material for: Effectiveness of public health measures and strategies to reduce risk of spread of respiratory pathogens at sporting mass gatherings: systematic literature review
Source: Front Public Health. 2026 Apr 8;14:1789413. doi: 10.3389/fpubh.2026.1789413 (PMC13099540; doi:10.3389/fpubh.2026.1789413)
Supplement: Supplementary file 6 [file Data_Sheet_5.pdf]

Supplemental File E (Table): Public Health Measures Implemented for Sporting Mass Gatherings Effectiveness Results and Public Health Measures Implemented for Each Event

Article	Sporting Mass Gathering Under Study	Public Health Measures Implemented	Effectiveness Analysis: Data Metric(s)	Effectiveness Analysis Conducted	Effectiveness Analysis: Results
**Al Musleh et al_2022[66]**	Asian Football Confederations League	Bubble<br><br>Country entry or exit testing<br><br>Enhanced hygiene practices – personal<br><br>Enhanced hygiene practices – venue<br><br>Isolation and quarantine measures<br><br>Limited capacity or no spectators<br><br>Mandatory vaccination<br><br>Mask-wearing<br><br>Minimizing physical interactions or physical distancing<br><br>Other<br><br>Routine testing<br><br>Temperature screening	Cases per x population / incident rate<br><br>Percent positive / positivity rate / attack rate<br><br>Total number or cases/incidents/events reported<br><br>Total number of tests conducted	Time trend for case detection across subgroups (local population; competition cohort)<br><br>Effectiveness of interventions within bubble<br><br>Distribution of cases across subgroups<br><br>Testing intensity and surveillance coverage	Average daily incident rate in Qatar public during tournament:<br>• 6.5 cases per 100,000 population;<br>• no spike detected within a week after tournament ends<br><br>Overall positivity rate for competition cohort: 0.15%<br><br>Total cases in competition cohort:<br>• 5 PCR positive cases reported (2 match officials; 3 local staff);<br>• 1 positive rapid antigen case in spectator cohort<br><br>Total tests conducted in competition cohort:<br>• 12,250 PCR tests conducted during tournament (within bubble);<br>• 10,320 rapid antigen tests in spectator cohort
**Al-Thani_2022[58]**	FIFA Arab Cup	Mandatory vaccination<br><br>Mask-wearing<br><br>Other	Cases per x population / incident rate<br><br>Percent positive / positivity rate / attack rate	Time trend for case detection across subgroups (host population; MG cohort)<br><br>Distribution of cases across diseases	Average incident rate of host population (COVID-19) at various time periods:<br>• one week before tournament started: 5 per 100,000 population<br>• Day one of tournament: 5.41 cases per 100,000 population<br>• End of tournament: 6 cases per 100,000 population<br>• Two weeks post tournament: 11.02 cases per 100,000 population

					Percent positive of MG participant cohort:<br>• 0.9% (COVID-19);<br>• 0.2% (influenza A/B);<br>• 0.6% (RSV)
**Ayala et al_2016**[54]	Superbowl	Enhanced surveillance	Total number of cases/incidents/events reported	Effectiveness of real-time monitoring / surveillance<br><br>Distribution of cases across diseases<br><br>Distribution of cases across events	Of 51 visits to clinic, 7 reports of ILI;<br><br>9 outbreak investigations all unrelated to MG;<br><br>Widespread influenza activity reported but activity was peaked before events began;<br><br>Event syndromic surveillance reported respiratory syndromes in 2/15 illnesses for Pro Bowl, 10/63 for NFL experience, 0/17 for Super Bowl
**Beebeejaun et al_2022**[33]	EURO 2020	Country entry or exit testing<br><br>Enhanced surveillance<br><br>Isolation and quarantine measures<br><br>Limited capacity or no spectators<br><br>Mandatory vaccination<br><br>Mask-wearing<br><br>Minimizing physical interactions or physical distancing<br><br>Routine testing	Total number of cases/incidents/events reported	Distribution of cases across events (with varied stadium capacities) / countries / event settings (official versus unofficial events)	A total of 9,612 cases of COVID-19 linked to EURO 2020 match attendance from 5 countries that reported data (England=6,376 cases; Scotland=2,632 cases; Finland=419 cases; Denmark=120 cases; Germany=15 cases)<br><br>England cases separated by games with differing stadium capacity (31 cases linked to games with 25% capacity; 247 cases linked to games with 50% capacity; 2,748 linked to games with 75% capacity)<br>Germany cases (n=15) reported at stadium with 20% capacity<br><br>Majority of cases in Scotland and Germany (73% and 89%) associated with unofficial EURO 2020 events
**Berland et al_2024**	Africa Cup of Nations (AFCON)	Country entry or exit testing<br><br>Enhanced hygiene practices – personal<br><br>Enhanced hygiene practices – venue<br><br>Enhanced surveillance<br><br>Isolation and quarantine measures<br><br>Mask-wearing	Percent positive / positivity rate / attack rate<br><br>Total number of cases/incidents/events reported<br><br>Total number of tests conducted	Time trend for case detection across subgroups (local population, neighbouring population, ACFON cohort)<br><br>Comparative epidemiology / trend analysis between event, background population and neighbouring populations	Percent positive, total number of cases<br>For AFCON testing:<br>• 309 (12.5%) were positive (all asymptomatic)<br><br>Total number of tests conducted<br>Active AFCON testing:<br>• 2481 tests performed<br><br>Percent change<br>Positivity rate overtime for AFCON:<br>• 26% week before AFCON

		Minimizing physical interactions or physical distancing<br><br>Other<br><br>Regular health survey checks or health monitoring<br><br>Routine testing	Percent growth / percent increases / percent change	Effectiveness of interventions (fan zone testing)<br><br>Testing intensity and surveillance coverage	• 12% first week of AFCON<br>• Below 1% for next 3 weeks<br>Positivity rate of passive testing programme in local population:<br>• 58% in early January 2022 (when AFCON testing began) and as Omicron emerged.<br>• During AFCON, positivity rate declined (28% in week 1, 6% in week 2, 0% until AFCON ended).<br><br>Number of cases steadily decreased during period of content both in MG participants and in local population<br><br>Comparison of Cameroon cases to that of Gabon and Nigeria;<br>• 1479 tests performed in Garoua city through passive screening (1 Nov 2021 - 31 Mar 2022); 1231 were from symptomatic persons; 266 positive (18%)<br>• Neighbouring countries - decline in cases was similar between three geographical contexts in subsequent seven weeks after MG.<br><br>Omicron was dominant variant and no significant difference in the proportion of variants detected by passive and active screening programmes
**Chowdhury et al_2023**[45,56]	Olympic Games (Tokyo 2020)	Bubble<br><br>Country entry or exit testing<br><br>Enhanced hygiene practices – personal<br><br>Enhanced hygiene practices – venue<br><br>Isolation and quarantine measures<br><br>Limited capacity or no spectators<br><br>Mask-wearing<br><br>Minimizing physical interactions or physical distancing	Cases per x population / incident rate<br><br>Percent growth / percent increases / percent change	Time trend for case detection across subgroups (Geographical regions)<br><br>Comparative epidemiology / trend analysis between geographical regions (Tokyo, Osaka, 5 other prefectures)<br><br>Associations between population density (high, low) and COVID-19 outbreak intensity	Monthly average incident rate of COVID-19:<br>• July (313.8 cases per 100,000 people in Tokyo, 121.59 cases per 100,000 people in Osaka, 88.75 cases per 100,000 people in five largest prefectures outside of Tokyo)<br>• August (839.06 cases per 100,000 people in Tokyo, 617.31 cases per 100,000 people in Osaka, 507.80 cases per 100,000 people in five largest prefectures outside of Tokyo)<br>• September (226.40 cases per 100,000 people in Tokyo, 348.66 cases per 100,000 people in Osaka, 258.68 cases per 100,000 people in five largest prefectures outside of Tokyo)<br><br>COVID-19 growth rate:

		Routine testing<br><br>Temperature screening			• August – negative growth in Tokyo and other prefectures;<br>• September - negative growth rate in Tokyo only;<br><br>Monthly growth rates:<br>• August on July (Tokyo – 185.25%; Osaka=407.68%; five largest prefectures outside Tokyo=472.14%)<br>• September on August (Tokyo – (-74.65%); Osaka=(-43.52%); five largest prefectures outside Tokyo=(-49.06%))<br>• September on July (Tokyo – (-27.69%); Osaka=186.74%; five largest prefectures outside Tokyo=191.46%)<br><br>No significant difference between distribution of confirmed cases between high and low density cities (Mann-Whitney U test, p<0.2)
**Cuschieri et al_2022**[34]	EURO 2020	Limited capacity or no spectators<br><br>Mask-wearing<br><br>Minimizing physical interactions or physical distancing<br><br>Routine testing<br><br>Temperature screening	Cases per x population / incident rate<br><br>Percent growth / percent increases / percent change	Time trend for case detection across subgroups (countries)<br><br>Comparative epidemiology / trend analysis between geographical regions	General increase in COVID-19 incident rates observed from start of tournament across in 7/11 host cities/regions<br><br>COVID-19 percent change:<br>• Netherlands (1,629% increase)<br>• Denmark (210% increase)<br>• Scotland (57% increase)<br>• England (382% increase)<br>• Germany (9% increase)<br>• Italy (104% increase)<br>• Russia (196% increase)<br>• Spain (135% increase)
**De Polo et al_2021**[63]	Cortina 2021 Alpine World Ski Championships	Bubble<br><br>Enhanced hygiene practices – personal<br><br>Enhanced hygiene practices – venue<br><br>Isolation and quarantine measures<br><br>Limited capacity or no spectators<br><br>Mask-wearing	Cases per x population / incident rate<br><br>Percent positive / positivity rate / attack rate<br><br>Total number of cases/incidents/events reported	Time trend for case detection<br><br>Comparative epidemiology / trend analysis between geographical regions<br><br>Effectiveness of interventions within bubbles	Comparison of weekly incidence between host population and similar municipality (Agordo) shows no difference<br><br>• Volunteers/organizers (blue bubble) had highest positivity rate of MG cohort (0.16%);<br>• Athletes (red bubble) had second highest positivity rate of MG cohort (0.09%)<br><br>Total number of positive COVID-19 cases: 22 cases

		Minimizing physical interactions or physical distancing<br><br>Regular health survey checks or health monitoring<br><br>Routine testing<br><br>Temperature screening	Total number of tests conducted	Distribution of cases across subgroups<br><br>Testing intensity and surveillance coverage	Total number of tests conducted: 19,031 antigen swabs were tested during event
**Dergaa et al_2022**[35]	Olympic Games (Tokyo 2020)	Bubble<br><br>Country entry or exit testing<br><br>Isolation and quarantine measures<br><br>Limited capacity or no spectators<br><br>Mask-wearing<br><br>Minimizing physical interactions or physical distancing<br><br>Routine testing<br><br>Temperature screening	Percent positive / positivity rate / attack rate<br><br>Total number of cases/incidents/events reported<br><br>Total number of tests conducted	Time trend for case detection in local population<br><br>Effectiveness of interventions in bubble<br><br>Distribution of cases by time period (Pre-Games; during; Post-Games) and event (Olympic Games, Paralympic Games)<br><br>Testing intensity and surveillance coverage	Total cases reported across Games: 863 cases (Olympic Games = 547 cases; Paralympic Games = 316 cases)<br>• Olympic Games (pre-Games=88; during Games=342; post Games=117)<br>• Paralympic Games (pre-Games=142; during Games=158; post Games=16)<br><br>Approx. number of new COVID-19 cases reported daily in Japan (host population)<br>• 1 July = 1,500 cases<br>• 24 July (peak) = 23,000 cases<br>• 6 Sept = 13,852 cases<br>• 22 October = 326 cases<br><br>Percent positive for individuals who left Japan and tested positive 14 days after departure: 0.0025%
**Dixon et al_2022**[67]	NCAA Men's Basketball Tournament	Bubble<br><br>Enhanced hygiene practices – venue<br><br>Isolation and quarantine measures<br><br>Mask-wearing<br><br>Minimizing physical interactions or physical distancing<br><br>Routine testing	Cases per x population / incident rate<br><br>Percent positive / positivity rate / attack rate<br><br>Total number of cases/incidents/events reported<br><br>Total number of tests conducted	Effectiveness of interventions in bubble<br><br>Distribution of cases across subgroups and by time period (prior to event, one week after event)<br><br>Comparative epidemiology / trend analysis between geographical regions<br><br>Testing intensity and surveillance coverage	National US incident rates:<br>• Prior to tournament: 16 per 100,000 population<br>• Prior to Final 4 Games: 20 per 100,000 population<br>• One week after tournament: 20 per 100,000 population<br>Indianapolis incident rates:<br>• Prior to tournament: 10 per 100,000 population<br>• One week after tournament: 19 per 100,000 population<br><br>Percent positive of MG cohort: 0.56%<br><br>Total number of positive COVID-19 cases: 15 cases

					• 8 cases were in Tier 1 (athletes/coaches/medical staff/officials)<br>• 3 cases were in Tier 2 (bus drivers/ event admin staff/security)<br>• 4 cases were in Tier 3 (event service staff)<br><br>Total number of tests conducted during MG: 28,311 tests during MG period
**Fulop et al_2022**[64]	International Swimming League 2020 Event	Bubble<br><br>Isolation and quarantine measures<br><br>Limited capacity or no spectators<br><br>Mask-wearing<br><br>Minimizing physical interactions or physical distancing<br><br>Other<br><br>Regular health survey checks or health monitoring<br><br>Routine testing	Percent positive / positivity rate / attack rate<br><br>Total number of cases/incidents/events reported<br><br>Total number of tests conducted	Effectiveness of interventions in bubble<br><br>Distribution of cases across subgroups<br><br>Comparative epidemiology / trend analysis between event and host country<br><br>Testing intensity and surveillance coverage	Positivity rate (COVID-19): 4.43%<br>Positivity rate of local staff exceeded national numbers (4.5-fold increase in COVID-19 compared to national results)<br><br>Total number of COVID-19 cases:<br>• 67 positive cases (63 considered positive after further examination; 3 athletes, 1 event staff, remaining positives were crew).<br>• No athletes in bubble tested positive (cases were identified on arrival testing)<br><br>Total number of COVID-19 tests conducted: 11,480 PCR tests
**Haddad et al_2017**[59]	6th Francophone Games	Enhanced hygiene practices – personal<br><br>Enhanced surveillance<br><br>Isolation and quarantine measures<br><br>Other	Total number of cases/incidents/events reported	Effectiveness of real-time monitoring / surveillance<br><br>Distribution of cases across syndromes	299 medical consultations reported at the event village clinic<br>• 21 febrile respiratory infections<br>• 7 other respiratory infections<br>• no swabs tests positive for H1N1<br>• no clustering observed
**Heese et al_2022**[52]	EURO 2020	Enhances surveillance<br><br>Limited capacity or no spectators<br><br>Mask-wearing<br><br>Other<br><br>Routine testing	Cases per x population / incident rate<br><br>Total number of cases/incidents/events reported	Time trend for case detection<br><br>Effectiveness of real-time monitoring / surveillance<br><br>Distribution of cases across geographic regions and subgroups (spectators,	COVID-19 daily 7 day incident rates at beginning of EURO 2020 (11 June):<br>• Munich – 22.3 cases per 100,000 people<br>• Bavaria – 22.8 cases per 100,000 people<br>• Germany – 19.3 cases per 100,000 people<br><br>COVID-19 daily 7 day incident rates at end of EURO 2020 surveillance period (25 July):<br>• Munich – 25.1 cases per 100,000 people

				imported cases, view parties)	• Bavaria – 13.6 cases per 100,000 people<br>• Germany – 15.0 cases per 100,000 people<br><br>Total number of COVID-19 cases:<br>• 5 cases of the 47,434 COVID-19 cases reported during time of event identified as spectators<br>• 10 notifications of imported COVID-19 cases who reported attending stadium matches<br>• 123 cases linked to EURO2020 viewing party but not affiliated with stadium attendance
**Huo et al_2023**[46]	Olympic Games (Beijing 2022)	Bubble<br><br>Country entry or exit testing<br><br>Enhanced hygiene practices – personal<br><br>Enhanced hygiene practices – venue<br><br>Isolation and quarantine measures<br><br>Mandatory vaccination<br><br>Mask-wearing<br><br>Minimizing physical interactions or physical distancing<br><br>Regular health survey checks or health monitoring<br><br>Routine testing	Percent positive / positivity rate / attack rate<br><br>Total number of cases/incidents/events reported<br><br>Total number of tests conducted	Effectiveness of interventions in bubble<br><br>Distribution of cases across subgroups<br><br>Distribution of cases by time period<br><br>Comparative epidemiology / trend analysis between event and host country<br><br>Testing intensity and surveillance coverage	Positivity rate:<br>• Airport screening=1.9% (60.6% of all positive cases detected at airport screening; 92% of all positive cases detected within 7 days or arrival to Beijing)<br><br>Total number of positive cases:<br>• 337 COVID-19 positive cases in event stakeholders;<br>• No spectators or other stakeholders outside bubble that worked on Games tested positive;<br>• 4 local residents tested positive<br><br>Total number of COVID-19 tests conducted: 1,859,423 PCR tests
**Kurland et al_2022**[68]	National Football League (NFL)	Enhanced hygiene practices – venue<br><br>Limited capacity or no spectators<br><br>Minimizing physical interactions or physical distancing<br><br>Other<br><br>Routine testing	Simple moving average of cases/rates	Time trend for case detection<br><br>Comparative epidemiology / trend analysis between geographical regions<br><br>Association between crowd size and COVID-19 incidence	Poisson mean tests for the 21-day simple moving day average (SMA), 14-day window in-county (cases: rate ratio 1·36 [95% CI 1·00–1·87], p<0·01) and 21-day SMA, 21-day window in-county (cases: rate ratio 1·49 [95% CI 1·21–1·83], p<0·0001, rates: rate ratio 1·50 [95% CI 1·26–1·78], p<0·0001)<br><br>Poisson mean tests for the 21-day SMA, 14-day window for surrounding counties (cases: rate ratio 1·31 [95% CI 1·00–1·72], p<0·01, rates: rate ratio 1·41 [95% CI 1·13–1·76],

					p<0·01), and 21-day SMA , 21-day window for surrounding counties (cases: rate ratio 1·37 [95% CI 1·14–1·65], p<0·01, rates: rate ratio 1·45 [95% CI 1·24–1·71], p<0·0001)<br><br>Games with fan attendance over 20,000 = 2.23 times the rate of spikes in COVID-19 compared to Games with less than 5,000 fan attendance
**Lim et al_2010**[36]	Asian Youth Games Singapore 2009	Isolation and quarantine measures<br><br>Mask-wearing<br><br>Other<br><br>Regular health survey checks or health monitoring<br><br>Temperature screening	Total number of cases/incidents/events reported	Effectiveness of real-time monitoring / surveillance<br><br>Distribution of cases across subgroups	Total number of cases:<br>• Suspected H1N1 = 66;<br>• Confirmed H1N1=6<br>Most cases were local workforce with community-acquired cases<br><br>No evidence of transmission within Games
**McCloskey et al_2014**[47]	Olympic Games (London 2012)	Enhanced surveillance<br><br>Other	Total number of cases/incidents/events reported	Effectiveness of real-time monitoring / surveillance<br><br>Distribution of cases across diseases	Total number of events reported:<br>• Respiratory (2 of 36 events reported in daily situation reports)<br>• Chickenpox (4 of 36 events reported in daily situation reports)<br>• Meningitis (1 of 26 events reported in daily situation reports)<br>Event syndromic surveillance did not report any outbreak related to the Games
**McCloskey et al_2024**[37]	Olympic Games (Tokyo 2020 and Beijing 2022)	Bubble<br><br>Enhanced hygiene practices – venue<br><br>Enhanced surveillance<br><br>Isolation and quarantine measures<br><br>Limited capacity or no spectators<br><br>Mask-wearing<br><br>Minimizing physical interactions or physical distancing	Percent positive / positivity rate / attack rate<br><br>Total number of cases/incidents/events reported<br><br>Total number of tests conducted	Time trend for case detection<br><br>Effectiveness of arrival testing and bubble screening<br><br>Distribution of cases across subgroups<br><br>Testing intensity and surveillance coverage	Positivity rates:<br>• Tokyo – Airport positivity rate=0.9%; bubble screening positivity rate= 0.02% (72% of positive cases detected within 14 days of arrival)<br>• Beijing – airport positivity rate = 1.94%; bubble screening positivity rate=0.01% (98% of positive cases detected within 14 days of arrival)<br><br>Total COVID-19 cases:<br>• Tokyo - 464 positive cases (33 athletes; 34 team officials)<br>• Beijing – 437 positive cases (98 athletes, 82 team officials)

		Other<br><br>Routine testing			Total number of tests conducted:<br>• Tokyo – approx. 676,000 tests conducted (42,971 airport tests, 633,818 screening tests)<br>• Beijing – 1.9 million tests (13,690 airport tests, 1,860,000 screening tests)
**Mikailova et al_2020[60]**	FIFA World Cup	Enhanced hygiene practices – venue<br><br>Enhanced surveillance<br><br>Isolation and quarantine measures<br><br>Other<br><br>Temperature screening	Total number of cases/incidents/events reported	Effectiveness of real-time monitoring / surveillance<br><br>Distribution of cases across diseases	Total number of cases:<br>• measles=341 cases;<br>• SARS=14 cases
**Morath et al_2022[69]**	Germany Volleyball Bundesliga 2020 Season	Isolation and quarantine measures<br><br>Mask-wearing<br><br>Minimizing physical interactions or physical distancing<br><br>Other<br><br>Routine testing	Total number of cases/incidents/events reported	Time trend for case detection<br><br>Effectiveness of real-time monitoring / surveillance<br><br>Distribution of cases across subgroup (players only)	Total number of cases:<br>• 15 players initially exposed<br>• 6 COVID-19 cases reported<br><br>Reporting by day from first match (day 1)<br>• Day 2: hypothesized infection of Player 1<br>• Day 6: player 1 is PCR positive; player 1-3 are quarantined (player 2 and 3 were previously showing symptoms)<br>• Day 8: Play 2 PCR positive<br>• Day 9: Player 3, 4 and 5 are PCR positive<br>• Day 36: Player 6 rapid antigen test positive<br>• Day 39: Player 6 PCR positive
**Murray et al_2020[70]**	2020 MLB Season	Enhanced hygiene practices – venue<br><br>Isolation and quarantine measures<br><br>Limited capacity or no spectators<br><br>Mask-wearing<br><br>Minimizing physical interactions or physical distancing<br><br>Other	Percent positive / positivity rate / attack rate<br><br>Total number of cases/incidents/events reported	Time trend for case detection<br><br>Distribution of cases across subgroups (teams, exposures)<br><br>Effectiveness of real-time monitoring/ surveillance	Attack rate:<br>• Team A = 29.4% (20 positive cases/68 exposed)<br>• Team B = 3.2% (1 positive cases/31 exposed)<br>• Team C = 0% (0 positive cases/38 exposed)<br><br>Total number of cases: (identified from outbreak investigation of 1 player from Team A)<br>• 21 COVID-19 cases (20 from team A and 1 from Team B)

		Regular health survey checks or health monitoring<br><br>Routine testing<br><br>Temperature screening			
**Nishino et al_2022**[65]	Volleyball Nations League	Bubble<br><br>Country entry or exit testing<br><br>Enhanced hygiene practices – personal<br><br>Enhanced hygiene practices – venue<br><br>Mask-wearing<br><br>Minimizing physical interactions or physical distancing<br><br>Regular health survey checks or health monitoring<br><br>Routine testing	Total number of cases/incidents/events reported<br><br>Total number of tests conducted	Effectiveness of interventions within bubble<br><br>Testing intensity and surveillance coverage	Total number of positive COVID-19 cases: 1 case<br><br>Total number of COVID-19 tests conducted:<br>• PCR = 2,250 tests;<br>• antigen = 7920 tests
**Pang et al_2017**[48]	Olympic Games (Beijing 2008)	Enhanced surveillance	Total number of cases/incidents/events reported	Effectiveness of real-time monitoring / surveillance<br><br>Distribution of cases across syndromes	Total number of ILI cases: 17,563 cases over reporting period of Games in host country<br><br>No early warning signal for influenza was triggered<br><br>No outbreaks of infectious disease reported during this time period
**Pauser et al_2021**[71]	2nd Division Professional Basketball League	Limited capacity or no spectators<br><br>Minimizing physical interactions or physical distancing<br><br>Other<br><br>Regular health survey checks or health monitoring<br><br>Temperature screening	Percent positive / positivity rate / attack rate<br><br>Percent growth / percent increases / percent change<br><br>Total number of cases/incidents/events reported	Effectiveness of mask-wearing behaviour<br><br>Distribution of cases across subgroups (mask wearing, non mask wearing)	Percent positive:<br>• 65% of participants tested positive (of the 90% of participants that were tested following a confirmed case)<br>• Of those that were positive (12 wore masks: 24 did not wear masks)<br>• Of those that were negative (14 wore masks; 4 did not wear masks)<br><br>Percent change: 37% reduction due to masking wearing (risk of transmission reduced from 83% (non-mask wearing) to 46% (mask wearing)

					Total number of cases of COVID-19 reported: 36 cases (3 cases were severe and required hospitalization)
**Riccardo et al_2022[53]**	EURO 2020	Enhanced surveillance<br><br>Limited capacity or no spectators<br><br>Mask-wearing<br><br>Minimizing physical interactions or physical distancing<br><br>Other<br><br>Routine testing	Total number of cases/incidents/events reported	Time trend of case detection<br><br>Distribution of cases across subgroups (age and gender)<br><br>Comparative epidemiology / trend analysis between geographical regions	Total number of COVID-19 cases reported: 137,993 cases reported during observation period (1 June to 31 July), mainly among younger males<br>• Daily range between 274 – 6404 cases<br>• Increasing trend in number of new infections with peak on 27 July<br>• 344 new cases identified with exposures linked to EURO 2020 matches but all cases linked to victory parties outside stadium<br>• 6 clusters identified throughout country with cases ranging from 3-100 cases, all linked to non stadium gatherings
**Robinson et al_2022[38]**	Golf Competitions (Dimension Data Pro Am and Bain's Whisky Cape Town Open)	Enhanced hygiene practices – personal<br><br>Enhanced hygiene practices – venue<br><br>Isolation and quarantine measures<br><br>Mandatory vaccination<br><br>Mask-wearing<br><br>Minimizing physical interactions or physical distancing<br><br>Other<br><br>Regular health survey checks or health monitoring<br><br>Routine testing	Cases per x population / incident rate<br><br>Total number of cases/incidents/events reported	Distribution of cases across subgroups<br><br>Comparative epidemiology / trend analysis between host city and event	National host city daily incident rates:<br>• Dimension Data Pro Am – 2 cases per 100,000 population<br>• Bain's Whisky Cape Town Open – 2 cases per 100,000 population<br><br>Total number of cases of COVID-19 reported: 3 tested positive during observation period (2 players, 1 caddie)
**Shimatani et al_2015[61]**	68th National Sports Festival	Enhanced surveillance	Total number of cases/incidents/events reported	Effectiveness of real-time monitoring / surveillance<br><br>Distribution across syndromes, surveillance	Total number of incidents reported:<br>• Official syndromic surveillance – 4 respiratory symptoms out of 7 incident reports<br>• Ambulance transfer syndromic surveillance – 0 definitive events out of 38 aberrations

				systems, and severity alert levels	• Pharmacy surveillances – 5 anti-varicella-Zoster drugs (medium level); 202 anti-varicella-Zoster drugs (medium level); 4 anti-influenza virus drugs (low level)<br>• (Nursery) School Absenteeism Surveillance system (absenteeism) – 4 acute respiratory symptoms and 0 ILI out of 22 aberrations<br>• (Nursery) School Absenteeism Surveillance system (suspension) – 17 respiratory illness (high level); 10 respiratory (medium level); 3 respiratory (low level)<br><br>No outbreaks reported in connection to MG
**Smith et al_2022**[55]	Multiple Events	Limited capacity or no spectators<br><br>Other	Total number of cases/incidents/events reported	Distribution of cases across events<br><br>Distribution of cases across infectious and acquisition windows	Total number of COVID-19 cases that attended an event during infectious period and during estimated period of acquiring COVID-19:<br>• International cricket: 123 (infectious period); 253 (acquired period)<br>• EURO 2020: 3036 (infectious period); 6376 (acquired period)<br>• Royal Ascot: 11 (infectious period); 39 (acquired period)<br>• Download Festival: 52 (infectious period); 65 (acquired period)<br>• Wimbledon Tennis: 299 (infectious period); 582 (acquired period)<br>• Goodwood Festival: 157 (infectious period); 168 (acquired period)<br>• The Open Golf: 100 (infectious period); 64 (acquired period)
**Sugishita et al_2023**[49]	Olympic Games (Tokyo 2020)	Enhanced surveillance<br><br>Limited capacity or no spectators	Total number of cases/incidents/events reported	Effectiveness of real-time monitoring / surveillance<br><br>Distribution across syndromes, disease and surveillance systems	Total number of incidents reports:<br>• Official notifiable disease surveillance – 0 respiratory cases of the 129 reported<br>• Sentinel surveillance sites -RSV most frequent with a peak of 8.93 cases reported per sentinel site (419 sites) in a week<br>• Syndromic surveillance - no reports made<br>• Cluster surveillance – 267 clusters of RSV; 77 clusters undiagnosed<br>• Ambulance transfer surveillance – 5 aberrant cases, no outbreaks observed

					• Tokyo infectious alert system -no reports during time period of interest<br>• Prescription and absenteeism surveillance – no aberrations reported<br><br>Surveillance confirmed no cases of infectious disease reported that required a public health response during MG period<br><br>None of the diseases reported through surveillance system were associated with the MG
**Tchounga et al_2025**[39]	Africa Cup of Nations (AFCON)	Country entry or exit testing<br><br>Enhanced hygiene practices – personal<br><br>Isolation and quarantine measures<br><br>Mask-wearing<br><br>Minimizing physical interactions or physical distancing<br><br>Other<br><br>Routine testing	Percent positive / positivity rate / attack rate<br><br>Total number of cases/incidents/events reported<br><br>Total number of tests conducted	Testing intensity and surveillance coverage<br><br>Distribution of cases by age, gender, country of spectator<br><br>Distribution of cases by fan zone	Total number of cases reported and percent positive<br>• 4,820 tests conducted<br>• The case detection rate was 40.1 (95% CI: 24.2–62.7) per 10,000 attendees.<br><br>• Of these participants tested, 4,671 had a negative test (96.8%)<br>• 148 (3.1%) had a positive Ag-RDT result<br>• 1 (0.1%) was indeterminate.<br><br>Of the positive tests, 64 consented to PCR testing and sharing of data and 19 of 64 (29.7%) were confirmed PCR-positive.<br><br>The Omicron variant (B.1.1.529) was found in all 11 samples successfully sequenced.<br><br>Total number of tests reported:<br>• 4,820 fan zone attendees were tested for SARS-CoV-2, including 1,228 (25.5%) fully vaccinated.
**Tsouros et al_2007**[32]	Olympic Games (Athens 2004)	Enhanced surveillance<br><br>Other	Total number of cases/incidents/events reported	Effectiveness of real-time monitoring / surveillance<br><br>Distribution of cases across syndromes, diseases and surveillance systems/sites<br><br>Testing intensity and surveillance coverage	Total number of incidents reported:<br>• Mandatory notification system - 443 cases total (tuberculosis = 77; varicella with complications = 3; pertussis = 6; mumps = 1 no SARS, influenza, measles)<br>• Lab reporting system - 94 cases total (1 parainfluenza; Mycoplasma pneumoniae = 18 cases)

					• Sentinel system - 3230 visits, 356 diseases or cases of syndromes reported (215 respiratory infections, 15 ILI, 6 chickenpox)<br>• Syndromic surveillance of emergency departments - 131054 visits, 11226 cases of syndromes reported (5551 respiratory infections)<br>• Syndromic surveillance of Olympic Venues - 8640 visits, 187 cases of syndromes reported (56 respiratory infections)<br>• Syndromic surveillance of cruise ships -1400 visits, 36 cases of syndromes reported (1 respiratory infection)
**Urashima et al_2022**[50]	Olympic Games (Tokyo 2020)	Bubble<br><br>Country entry or exit testing<br><br>Enhanced hygiene practices – personal<br><br>Enhanced hygiene practices – venue<br><br>Limited capacity or no spectators<br><br>Minimizing physical interactions or physical distancing<br><br>Other<br><br>Routine testing	Total number of cases/incidents/events reported<br><br>Total number of tests conducted	Effectiveness of interventions in bubble<br><br>Distribution of cases across subgroups<br><br>Testing intensity and surveillance coverage	Total number of COVID-19 cases reported: 13 positive cases<br>• 3 athletes positive<br>• 8 officials<br>• 2 no response<br><br>Total number of PCR COVID-19 tests conducted: 12,370 tests conducted in total<br>• 10,229 tests conducted in Village medical clinic (all positive cases found in Village clinic)<br>• 2,141 tests at other medical clinics on site
**White et al_2018**[62]	8th Micronesian Games	Enhanced surveillance<br><br>Other	Total number of cases/incidents/events reported	Time trend of case detection<br><br>Effectiveness of real-time monitoring / surveillance<br><br>Distribution of cases across syndromes and diseases<br><br>Testing intensity and surveillance coverage	Total number of incidents reports: 5,640 encounter cases and 408 syndrome cases total from 11 sentinel sites<br>• ILI most prominent syndrome (55%, n=225)

**Xiong et al_2023**[51]	Olympic Games (Beijing 2022)	Bubble<br><br>Enhanced hygiene practices – personal<br><br>Isolation and quarantine measures<br><br>Mandatory vaccination<br><br>Mask-wearing<br><br>Minimizing physical interactions or physical distancing<br><br>Other<br><br>Regular health survey checks or health monitoring<br><br>Routine testing	Total number of cases/incidents/events reported	Effectiveness of interventions within bubble<br><br>Time trend for case detection<br><br>Distribution of cases by setting (initial screening, within bubble, local population) and over time (first 14 days)<br><br>Comparative epidemiology / trend analysis between settings (host population and event)<br><br>Projected scenario modelling	Total number of COVID-19 cases in bubble: 280 positive cases enrolled in study<br>• 93 cases (33.2%) identified through initial screening<br>• 274 cases (97.9% were identified within first 14 days of bubble/ considered imported cases)<br>• 6 cases (2.1%) were infected within bubble<br><br>Total number of COVID-19 cases outside bubble (local host population): 108 residents (cases were not associated with Games)<br><br>Modelling of a semi-closed loop estimates an increase in cases to 1,137 if identified quickly and quarantine measures implemented (3 days later) and 5.530 if identified later and quarantine measures implemented after 9 days
