# Supplementary material for: Effectiveness of public health measures and strategies to reduce risk of spread of respiratory pathogens at sporting mass gatherings: systematic literature review
Source: Front Public Health. 2026 Apr 8;14:1789413. doi: 10.3389/fpubh.2026.1789413 (PMC13099540; doi:10.3389/fpubh.2026.1789413)
Supplement: Supplementary file 7 [file Data_Sheet_6.pdf]

## Supplemental File F (Table): Effectiveness of Protocols to Mitigate Respiratory Disease Spread by MG

| Article | Sporting Mass Gathering Under Study | Effectiveness Determination | Criteria and Rationale |
|---|---|---|---|
| **Al Musleh et al_2022**[66] | Asian Football Confederations League | Effective | Low number of cases / positivity rate (6 cases; 0.15% within bubble)<br><br>High number of tests conducted<br><br>No intra-event transmission<br><br>No outbreaks or clusters detected during the event<br><br>No community spread linked to the event |
| **Al-Thani_2022**[58] | FIFA Arab Cup | Effective | Low positivity rate across diseases<br><br>No intra-event transmission<br><br>No community spread directly linked to the event (however slight increase in community two weeks post event, but stable rates throughout event) |
| **Ayala et al_2016**[54] | Superbowl | Indeterminate | Low number of signals, cases, syndromes identified (e.g. 7 cases of ILI across 51 site visits)<br><br>No outbreaks or clusters linked to the event<br><br>Distribution of cases across events shows minimal transmission<br><br>Functioning event-based surveillance system in place |
| **Beebeejaun et al_2022**[33] | EURO 2020 | Mixture (due to multiple events) | Low number of cases in stadiums<br><br>Minimal intra-event transmission for those attending matches (within stadiums); large intra-event transmission for those at unofficial watch parties, victory parties and other related gatherings<br><br>Outbreaks and clusters linked to some matches with higher capacity<br><br>*should be noted that most cases identified were linked to unofficial events |
| **Berland et al_2024** | Africa Cup of Nations (AFCON) | Effective | Relatively low cases / positivity rate in MG cohort (12.5% for all weeks, however on week-by-week basis decreased as event went on)<br><br>No outbreaks or clusters linked to event<br><br>Community cases trended stable or decreased in cases as event went on<br><br>High number of tests conducted |

| | | | |
|---|---|---|---|
| **Chowdhury et al_2023**[45,56] | Olympic Games (Tokyo 2020) | Indeterminate | No direct link between rises in community cases and Games (with possible alternative explanations provided for increases in cases in communities)<br><br>Increases in cases in all communities under study (not just Tokyo where Games occurred suggesting nationwide rises)<br><br>Spikes of outbreaks or clusters during Games however not clearly linked to Games |
| **Cuschieri et al_2022**[34] | EURO 2020 | Non effective | Increases in cases during and after events<br><br>Percent change in incidence ranged between countries but mostly large positive change (indicating sharp increase in cases) |
| **De Polo et al_2021**[63] | Cortina 2021 Alpine World Ski Championships | Effective | Low number of cases reported / low positivity rate (22 cases across cohort; highest positivity rate was 0.16 positivity rate)<br><br>High number of tests conducted (>19,000)<br><br>No increase in incidence in local population compared to neighbouring areas |
| **Dergaa et al_2022**[35] | Olympic Games (Tokyo 2020) | Effective | Relatively low number of cases across Games (863 cases across Olympic and Paralympic pre, during and post Games)<br><br>Low positivity rate for participants rate upon ending their Games experience and exiting Japan (0.0025%)<br><br>No evidence of intra-event transmission<br><br>Local population cases and trends appear to not be linked to Games |
| **Dixon et al_2022**[67] | NCAA Men's Basketball Tournament | Effective | Low number of cases / low percent positive (15 cases; 0.56%)<br><br>High number of tests conducted (>28,000)<br><br>No evidence of intra-event transmission<br><br>Small increase in cases in local population but align with national incident trends |
| **Fulop et al_2022**[64] | International Swimming League 2020 Event | Effective | Low-moderate positivity rate (4.43%), all cases detected on arrival<br><br>High number of tests conducted (>11,000)<br><br>No intra-event transmission |
| **Haddad et al_2017**[59] | 6th Francophone Games | Effective | Low number of significant public health incidents / low number and proportion of respiratory cases (<10%)<br><br>No outbreaks or clusters detected during the Games<br><br>Functioning syndromic surveillance system in place |

| | | | |
|---|---|---|---|
| **Heese et al_2022**[52] | EURO 2020 | Effective | Low number of stadium confirmed cases (5/47,434 cases)<br><br>Limited intra-event transmission within stadium<br><br>Functioning event-based surveillance system in place |
| **Huo et al_2023**[46] | Olympic Games (Beijing 2022) | Effective | Low number of cases reported<br><br>High number of tests conducted<br><br>No evidence of significant spread from Games population to local population<br><br>Rapid identification and response, limiting risk of escalation |
| **Kurland et al_2022**[68] | National Football League (NFL) | Non effective | Significant increase in cases after event<br><br>Crowd size linked to higher post-event increases<br><br>Presence of outbreaks detected |
| **Lim et al_2010**[36] | Asian Youth Games Singapore 2009 | Effective | Low number of cases detected at Games<br><br>No intra-event transmission<br><br>No outbreak detected within Games, possible outbreak traced in community (local staff) |
| **McCloskey et al_2014**[47] | Olympic Games (London 2012) | Effective | Low number of significant public health incidents<br><br>No outbreaks detected during the Games<br><br>Functional syndromic and event-based surveillance in place |
| **McCloskey et al_2024**[37] | Olympic Games (Tokyo 2020 and Beijing 2022) | Effective | Low number of cases detected (positivity rate 0.02% for Tokyo and 0.01% for Beijing)<br><br>High number of tests conducted<br><br>No evidence of significant spread from Games population to local population<br><br>No major outbreaks or clusters reported connect to the Games<br><br>Rapid identification and response, limiting risk of escalation |
| **Mikailova et al_2020**[60] | FIFA World Cup | Indeterminate | A number of significant public health incidents reported, however no mention of proportion of cases<br><br>No mention on intra-event transmission<br><br>No comparison of cases from event compared to community<br><br>Functional surveillance in place |

| | | | |
|---|---|---|---|
| **Morath et al_2022**[69] | Germany Volleyball Bundesliga 2020 Season | Indeterminate | Small outbreak (cluster)<br><br>Instances of intra-event transmission detected, however no ongoing chain reported or link to wider spread (no spread to wider cohort) |
| **Murray et al_2020**[70] | 2020 MLB Season | Indeterminate | Rapid identification and response, limiting risk of escalation<br><br>Small outbreak (cluster)<br><br>Instances of intra-event transmission detected, however no ongoing chain reported or link to wider spread (1 instance of spread to Team B, none to Team C) |
| **Nishino et al_2022**[65] | Volleyball Nations League | Effective | Rapid identification and response, limiting risk of escalation<br><br>Low number of cases detected (1 case)<br><br>High number of tests conducted (>10,000; multiple tests per participant throughout tournament)<br><br>No outbreaks or further spread detected |
| **Pang et al_2017**[48] | Olympic Games (Beijing 2008) | Effective | High number of ILI cases triggered in community however no influenza early warning signal triggered<br><br>No outbreaks detected during the Games<br><br>Functional syndromic and event-based surveillance in place |
| **Pauser et al_2021**[71] | 2nd Division Professional Basketball League | Non effective | High number of cases detected (65% positivity rate)<br><br>Further spread detected (multiple cases and 3 hospitalizations)<br><br>*should be noted that not all participants wore masks (intervention with mixed implementation). Of those that did, cases were lower |
| **Riccardo et al_2022**[53] | EURO 2020 | Indeterminate | Low number of new cases directly linked to event (0.25%; 344 or 137,993 cases)<br><br>Clusters detected (6 separate clusters)<br><br>Functional syndromic and event-based surveillance in place<br><br>*should be noted, no cases linked to stadium event (actual matches), but to viewing and victory parties where measures were less strict/ unenforced (relied on local public health measures only) |
| **Robinson et al_2022**[38] | Golf Competitions (Dimension Data Pro Am and Bain's Whisky Cape Town Open) | Effective | Low number of cases<br><br>No outbreaks or clusters detected during the events |

| | | | |
|---|---|---|---|
| **Shimatani et al_2015[61]** | 68th National Sports Festival | Effective | No cases or alerts linked to the event<br><br>No outbreaks or clusters detected during the event<br><br>Functional syndromic and event-based surveillance in place |
| **Smith et al_2022[55]** | Multiple Events | Mixture (due to multiple events) | Some events reported minimal transmission (low case numbers), others reported major spikes associated with the event<br><br>Functional surveillance in place (NHS Test and Trace) |
| **Sugishita et al_2023[49]** | Olympic Games (Tokyo 2020) | Effective | No cases or alerts linked to the event<br><br>No outbreaks or clusters detected that required a public health response<br><br>Functional syndromic surveillance in place |
| **Tchounga et al_2025[39]** | Africa Cup of Nations (AFCON) | Effective | Low number of cases detected (3.1% positivity)<br><br>No outbreaks or clusters detected during the event |
| **Tsouros et al_2007[32]** | Olympic Games (Athens 2004) | Effective | Low number/ proportion of respiratory cases from all reported syndromes<br><br>No outbreaks detected during the event that required public health response<br><br>Functional syndromic and event-based surveillance in place |
| **Urashima et al_2022[50]** | Olympic Games (Tokyo 2020) | Effective | Low number of cases (13 cases, 0.11%)<br><br>No outbreaks or clusters detected during the event<br><br>Functional surveillance in place |
| **White et al_2018[62]** | 8th Micronesian Games | Effective | Low number of cases<br><br>No outbreaks or clusters detected during the event<br><br>Functional syndromic and event-based surveillance in place |
| **Xiong et al_2023[51]** | Olympic Games (Beijing 2022) | Effective | Minimal intra event transmission<br><br>No outbreaks in host population that are linked to the event (ongoing outbreak in host population)<br><br>Rapid identification and response, limiting risk of escalation |
